# Microbiome structure in large pelagic sharks with distinct feeding ecologies

**DOI:** 10.1186/s42523-022-00168-x

**Published:** 2022-03-04

**Authors:** Zoe A. Pratte, Cameron Perry, Alistair D. M. Dove, Lisa A. Hoopes, Kim B. Ritchie, Robert E. Hueter, Chris Fischer, Alisa L. Newton, Frank J. Stewart

**Affiliations:** 1 Department of Microbiology & Cell Biology, Montanta State University, 621 Leon Johnson Hall, Bozeman, MT 59717 USA; 2 Center for Microbial Dynamics and Infection, School of Biological Sciences, Georgia Institute of Technology, Atlanta, GA USA; 3 Georgia Aquarium, Atlanta, GA USA; 4 University of South Carolina Beaufort, Beaufort, SC USA; 5 Present Address: OCEARCH, 1790 Bonanza Drive, Park City, UT USA; 6 Center for Shark Research, Mote Marine Laboratory, 1600 Ken Thompson Parkway, Sarasota, FL USA; 7 Disney’s Animals, Science and Environment, 1200 N. Savannah Circle East, Bay Lake, FL USA

**Keywords:** Elasmobranch, Whale shark, White shark, Tiger shark, Bacteria, Photobacterium

## Abstract

**Background:**

Sharks play essential roles in ocean food webs and human culture, but also face population declines worldwide due to human activity. The relationship between sharks and the microbes on and in the shark body is unclear, despite research on other animals showing the microbiome as intertwined with host physiology, immunity, and ecology. Research on shark-microbe interactions faces the significant challenge of sampling the largest and most elusive shark species. We leveraged a unique sampling infrastructure to compare the microbiomes of two apex predators, the white (*Carcharodon carcharias*) and tiger shark (*Galeocerdo cuvier*), to those of the filter-feeding whale shark (*Rhincodon typus*), allowing us to explore the effects of feeding mode on intestinal microbiome diversity and metabolic function, and environmental exposure on the diversity of microbes external to the body (on the skin, gill).

**Results:**

The fecal microbiomes of white and whale sharks were highly similar in taxonomic and gene category composition despite differences in host feeding mode and diet. Fecal microbiomes from these species were also taxon-poor compared to those of many other vertebrates and were more similar to those of predatory teleost fishes and toothed whales than to those of filter-feeding baleen whales. In contrast, microbiomes of external body niches were taxon-rich and significantly influenced by diversity in the water column microbiome.

**Conclusions:**

These results suggest complex roles for host identity, diet, and environmental exposure in structuring the shark microbiome and identify a small, but conserved, number of intestinal microbial taxa as potential contributors to shark physiology.

**Supplementary Information:**

The online version contains supplementary material available at 10.1186/s42523-022-00168-x.

## Introduction

The factors shaping animal health remain critically understudied for many ecologically important species. Notable among these are the large migratory sharks such as the apex-predatory white shark (*Carcharodon carcharias*) and filter-feeding whale shark (*Rhincodon typus*). As predators, sharks structure marine food webs, with shark removal having significant effects on lower trophic levels [1, 2]. Sharks also have high economic and cultural value—shark fisheries and tourism inject over $1.3 billion into the global economy [3, 4]. By virtue of unique developmental and immune properties, sharks are targets of biomedical research to understand adaptive and acquired immunity [5, 6], tooth regeneration [7], and anti-infection potential [8, 9]. Notably, sharks appear to have a high capacity for wound healing without infection [10, 11], a property potentially linked to antibiotic-producing bacteria in the epidermal mucus [12]. Despite their ecological, societal, and biomedical significance, shark populations are declining worldwide due to habitat degradation, overfishing, and other stressors [13, 14]. The International Union for Conservation of Nature (IUCN) lists 143 shark species (30% of all shark species) as “endangered” or “critically endangered”, “near threatened”, or “vulnerable”—these include the white shark (vulnerable) and whale shark (endangered). Understanding the health of these and other shark species has therefore emerged as a research priority.

Microorganisms on and in the shark body (the microbiome) may play important roles in shark biology but remain virtually unexplored. In other animals, a healthy microbiome has been linked to maturation of the immune system, pathogen suppression, maintenance of blood chemistry, toxin degradation, and the provisioning of essential nutrients [15, 16]. Microbiome members interact with circulating immune cells and lymphoid tissues, stimulating immune and brain development, affecting cognition, behavior, and even altering mood [17, 18] and possibly influencing mating [19 but see 20]. The microbiome, which may contain representatives from all three domains of life plus viruses, is shaped by a variety of factors, including diverse aspects of host physiology and the surrounding environment. These factors differ among body niches. In vertebrates, diet strongly influences the gut microbiome but has limited influence on the skin microbiome, which is more strongly influenced by host physiology and environmental conditions, with the latter including the composition of the surrounding microbiome [21, 22]. Determining the diversity and biochemical function of the microbiome and how these change with host and environmental variation is critical for understanding the overall importance of microorganisms to animal health and ecology.

With the growing need to understand shark health and ecology, researchers are beginning to characterize the shark microbiome. Preliminary work has focused mainly on skin-associated microbes, as the skin niche is relatively easy to sample compared to internal body compartments. A recent analysis showed that the skin microbiome of cartilaginous fishes, including the whale shark and two other shark species, showed compositional patterns suggestive of host-microbiome co-evolution, so-called ‘phylosymbiosis’ [23]. However, other factors such as variation in the seawater microbiome likely also strongly affect the external microbiome of sharks [24, 25], suggesting the potential for microbiome reorganization in response to environmental change (e.g., during migrations, or transitions from wild to managed care in aquaria). In contrast, the intestinal/fecal microbiome of sharks remains essentially uncharacterized. This is particularly true for large, migratory species for which obtaining fecal samples from live individuals requires either a highly-specialized sampling infrastructure, or happenstance. The only community-level analysis of shark intestinal microbiomes examined two individuals from each of three species, identifying a small number of shared (core) microbiome members [26]. However, the functional contribution of these microbes to shark health is unclear, as are the factors causing microbiome differences among host species, individuals, or body site niches.

As is true in other vertebrate microbiomes [27], shark intestinal microbiomes are likely shaped by differences in host diet, feeding behavior, and digestive anatomy. Most sharks are predators that can be either generalists, specialists, or opportunistic scavengers. The white shark (*C. carcharias*), for example, has a diverse diet, including fish, sea turtles, sea birds, and marine mammals, either live or dead [28, 29]. While *C. carcharias* and other apex predatory sharks are thought to feed infrequently on large meals high on the trophic level [30], few studies have described feeding behavior in the wild [31], and estimates of metabolic demand suggest that the white shark feeds more frequently than previously assumed [32].

In contrast, the filter-feeding behavior of whale (*Rhincodon typus*), basking (*Cetorhinus maximus*), and megamouth (*Megachasma pelagios*) sharks more closely resembles that of baleen whales. Filter-feeding sharks are thought to be primarily carnivorous at a low trophic level, consuming zooplankton, jellyfish, or small fishes of a narrow size range [33–35]. However, seagrass has been found in *R. typus* stomachs, and recent isotope data raise the possibility that plant material can account for roughly half of an individual’s diet, although whether this is incidental or targeted ingestion remains unresolved [36]. Isotope analysis also suggests that *R. typus* fast periodically [36], but they can also feed for hours (average: 7.5) each day at seasonal aggregation sites that host temporal spikes in prey abundance [37]. These observations highlight unresolved questions about the diet ecology of filter-feeding sharks.

There are also uncertainties regarding the intestinal physiology of filter-feeding sharks. Descriptions of the shark digestive system have focused almost exclusively on apex predatory species, which appear well adapted for carnivory. These species have an acidic, muscular J-shaped stomach for biochemical breakdown of proteins, a relatively short colon, and a unique spiral intestine that slows digestion time and maximizes nutrient absorption [38]. How these organs differ between filter-feeding and apex predatory sharks is not well documented. Necropsy reports have noted that *R. typus* have a more flaccid bag-like stomach compared to the strong muscular organ typical of macropredatory species and have a compact but otherwise typical spiral intestine (A. Dove, personal communication). However, studies have yet to describe the physiology of these organs in filter-feeding sharks, nor to explore the functional roles of microorganisms in shark digestion.

Variation in shark diet, behavior, and digestive physiology among trophic levels raises the possibility of a dynamic microbe-host relationship both within a shark species (e.g., during fasting versus non-fasting) and between species with different feeding modes. We explore this possibility by comparing the microbiomes of healthy individuals of three large pelagic shark species with distinct feeding strategies: the apex predatory white shark (*Carcharodon carcharias*) and tiger shark (*Galeocerdo cuvier*), and the filter-feeding whale shark (*Rhincodon typus*). Sampling from live animals was possible through a combination of ship-based line capture of wild individuals (in partnership with shark tracking specialists at OCEARCH), capture-free sampling via divers/swimmers, and collections from animals under managed care at Georgia Aquarium. With samples from six geographic locations over two years, we assess microbiome taxonomic composition across body niches (gill, skin, cloaca, feces), quantify the role of environmental and host factors (seawater microbiome composition, host species, body site) in structuring microbiome composition, and compare fecal microbiome metagenomes to assess potential variation between apex predatory sharks versus filter-feeding lifestyles.

## Results

### Sample collection

Sampling large sharks presents substantial difficulty associated with finding and safely handling live individuals. Sampling of *Carcharodon carcharias* and *Galeocerdo cuvier* was possible by a partnership with OCEARCH, a non-profit organization that facilitates studies of large marine animals with expertise in shark tracking, handline capture of large individuals, and shark handling on a semi-submerged research platform. All *C. carcharias* and *G. cuvier* samples were taken from wild adult and sub-adult animals (fork length: 204–421 cm) sampled using the OCEARCH infrastructure*,* as described in [39, 40]. *Carcharodon carcharias* and *Galeocerdo cuvier* samples span four OCEARCH expeditions in the Atlantic Gulf Stream, the Southeast (Florida to South Carolina coast), and Nova Scotia. *Carcharodon carcharias* and *Galeocerdo cuvier* were sampled in the same time period only during the NASFA (Northwest Atlantic Shared Foraging Area) expedition. Sampling of *Rhincodon typus* was facilitated by access to managed individuals at Georgia Aquarium and wild individuals sampled by divers/swimmers in the Maldives, Tanzania, and St. Helena (South Atlantic). In total, the sample set represents 7, 22, and 37 individuals of *Galeocerdo cuvier*, *Carcharodon Carcharias*, and *Rhincodon typus,* respectively, with samples including swabs of the gill, dorsal skin, and cloaca, and fecal matter collected either via sterile rubber catheter (*C. carcharias)* or directly from the water column after defecation (*Rhincodon typus*). Sample collections are summarized in Additional file 1: Table S1 and Additional file 1: Table S2 lists metadata and shark names for all OCEARCH samples. Because field conditions and operational circumstances varied for each collection, not all sample types are represented for each individual.

### Shotgun metagenomics

**Fig. 1:**
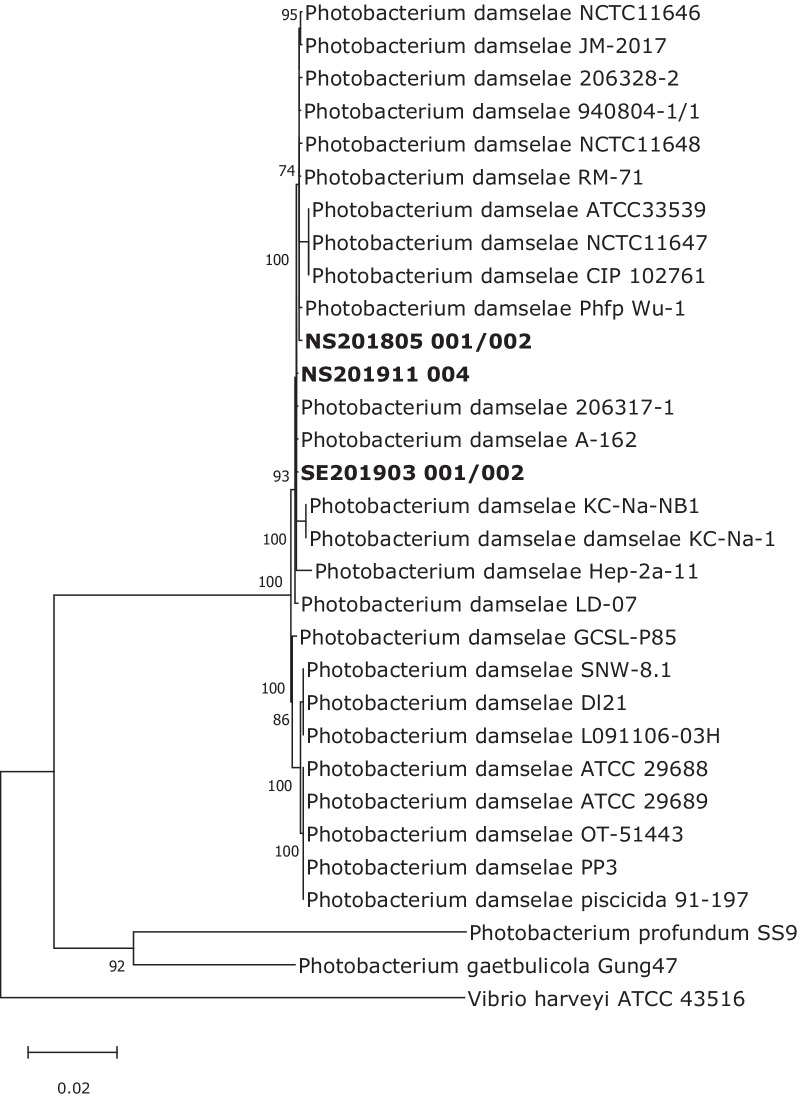
Maximum Likelihood phylogeny using a Dayhoff matrix-based model for three *Photobacterium* Metagenome Assembled Genomes (MAGs) from *Carcharodon carcharias* (bolded), along with 26 closely related genomes. Phylogeny was constructed on the basis of 91 concatenated single-copy marker genes identified by MiGA using Hidden Markov Models (HMMs) via HMMER3 with default settings. Marker genes were aligned using clustalW and then concatenated in MEGAX, numbers indicate bootstrap values (500 iterations)

Shotgun metagenomic sequencing was performed to assess microbial functional capabilities in seven fecal samples from *C. carcharias* and one from *R. typus* (DNA from the other four *R. typus* fecal samples was not of sufficient integrity for metagenomic analysis). Library size ranged from 5,521,299 to 15,968,373 paired end reads, with one library also containing 3,496,507 long reads over 1000 bp. Additional metagenome assembly statistics are in Additional file 1: Table S3. Initially, each fecal metagenome was analyzed independently. We recovered high-quality MAGs (metagenome assembled genomes) representing diverse bacteria classified as Clostridia, Fusobacterium, Campylobacter, *Photobacterium damselae,* and *Bacteriodes fragillis* (Additional file 1: Table S4), reflecting the composition determined by the amplicon analysis. All three high-quality *Photobacterium damselae* MAGs clustered within the *P. damselae damselae* subspecies (Fig. 1), with an average nucleotide similarity (ANI) of above 98% with other *P. damselae* isolates from various marine teleost fish species (Additional file 1: Table S5).

**Fig. 2:**
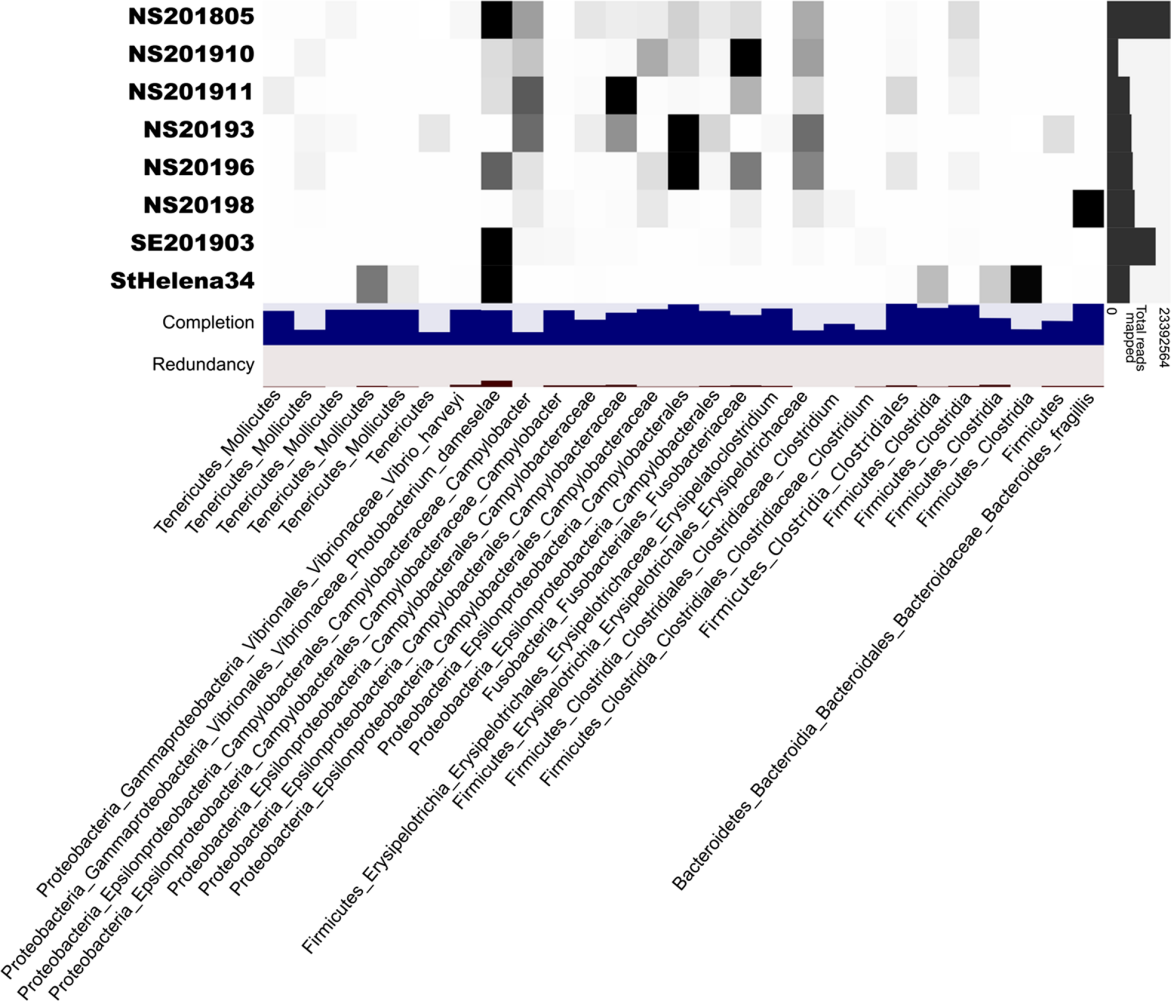
Mean depth of coverage across 27 co-assembled bins from one *Rhincodon typus* (StHelena34) and seven *Carcharodon carcharias* fecal samples. Bins were identified by MaxBin2.0 and manually refined in Anvi'o, and represent Metagenome Assembled Genomes (MAGs). Taxonomy is given to the lowest significant taxonomic resolution, as identified by MiGA. Completion estimates the percentage of the true bacterial genome represented in the MAG, and redundancy estimates the number of genes originating from another organism (contamination)

Based on community compositional similarity, sequences from all eight metagenomes were co-assembled and compared in Anvi'o [41]. Only one *P. damselae* MAG was produced, suggesting that all eight sharks share a highly similar strain. However, assembly of *P. damselae* genomes can be hindered by a high numbers of insertion sequences [42], raising the possibility that additional MAGs may be present in the community but not detected in the co-assembly. Regardless, *P. damselae* was found in high relative abundance in the fecal metagenomes of one *R. typus* individual and three *C. carcharias* individuals and in moderate abundance in two other *C. carcharias* individuals (Fig. 2). Interestingly, one *C. carcharias* fecal metagenome not dominated by *P. damselae* was dominated by *Bacteroides fragillis*. This taxon was not detected in any other fecal metagenomes.

**Fig. 3:**
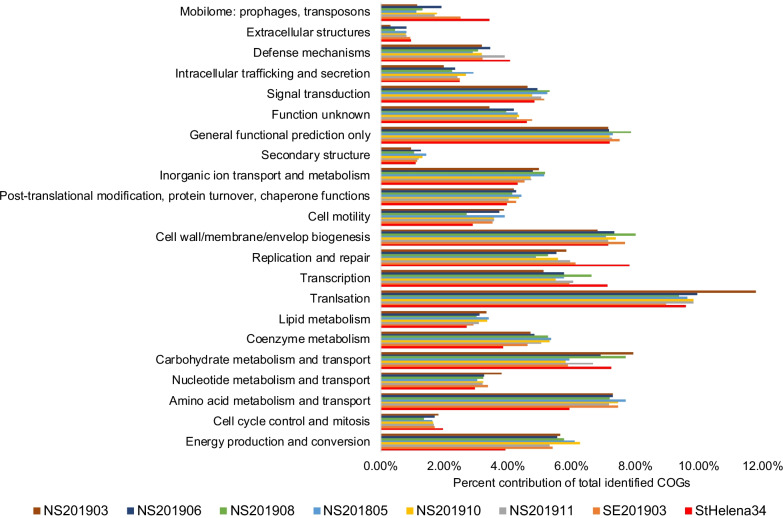
Relative abundance of Clusters of Orthologous Groups (COGs) for each fecal metagenome; one *Rhincodon typus* (StHelena34) and seven *Carcharodon carcharias*. The COG categories "RNA processing and modification", "Chromatin Structure and dynamics", and "Cytoskeleton and Nuclear structure" are not shown, as they contributed less than 1% to any one metagenome

**Fig. 4:**
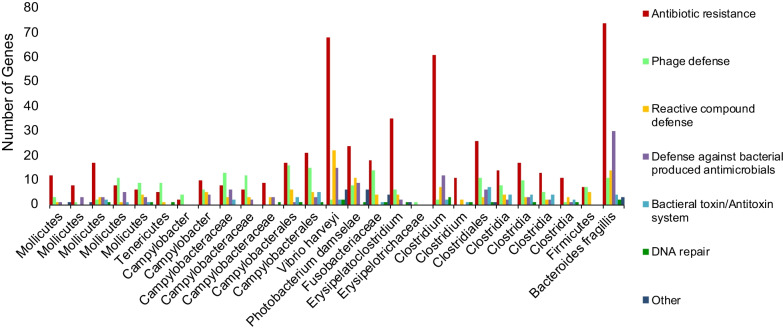
Categorical breakdown of Clusters of Orthologous Groups (COG) genes that classified as "defense mechanisms" from 27 Metagenome Assembled Genomes (MAGs) from one *Rhincodon typus* and seven *Carcharodon carcharias* fecal samples. Genes associated with antibiotic resistance are the most abundant of all defense mechanism categories

The relative abundances of genes assigned to Clusters of Orthologous Groups (COGs) were evaluated for entire fecal metagenomes (Fig. 3) and for individual MAGs (Additional file 1: Fig. S1). Because only one quality fecal metagenome was obtained from *R. typus,* it was not possible to statistically identify COGs differentially abundant between *R. typus* and *C. carcharias*. Nonetheless, the datasets preliminarily indicate that COG categories "Amino acid metabolism and transport" and "Energy production and conversion" are depleted in *R. typus* compared to *C. carcharias* (Fig. 3). Finally, for each MAG, the COG category "Defense mechanisms" was further evaluated to gain insight into the nature of microbial interactions within the shark fecal microbiome. An overwhelming majority of defense mechanism genes (508 total genes) are putatively involved in antibiotic resistance (Fig. 4). These antibiotic resistant genes were distributed across MAGs, and were not specific to any one bacterial taxon. Defense against phages (187 genes), reactive compounds (119 genes), and bacteria-produced antimicrobials (116 genes) were the next most abundant defense mechanism categories, followed by bacterial toxin/antitoxin systems (43 genes) and DNA damage repair (20 genes).

### 16S rRNA gene amplicons

**Fig. 5:**
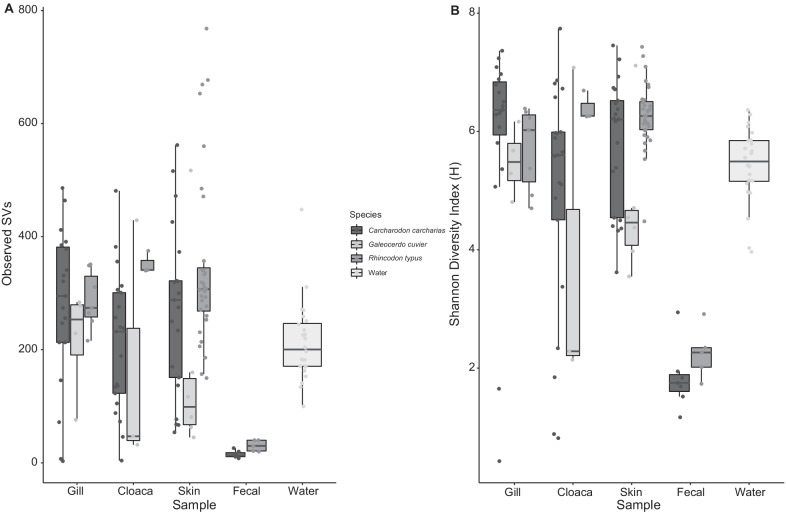
Microbiome alpha diversity across body sites and surrounding water associated with *Carcharodon carcharias*, *Galeocerdo cuvier,* and *Rhincodon typus*. Alpha diversity is shown as Observed Sequence Variants (SVs, panel **A**) and Shannon Diversity (H, panel **B**), with box plots displaying the median (bold line), 25th-75th percentiles, and whiskers showing a maximum 1.5 × IQR (interquartile range). Sample details and numbers are given in Additional file 1: Table S1 and Additional file 1: Table S2

**Fig. 6:**
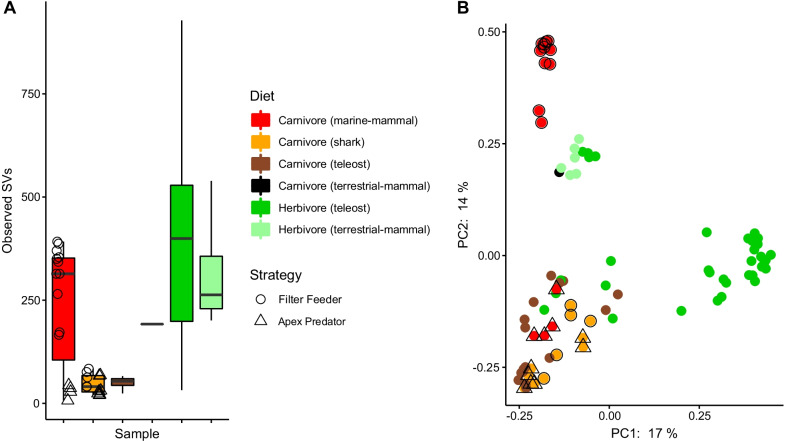
Alpha and beta diversity metrics comparing shark fecal samples (this study) to other teleost fish (Lemon-peel Angelfish, Peacock Grouper, Arc-eye Hawkfish, Lionfish, Pale-nose Parrotfish, Farmer Fish, data from Pratte et al. [22]) and mammals (Sei Whale, Bottlenose Dolphin, Rabbit, White-tailed Deer, Fisher-Cat, Humpback Whale, Hippo, Beluga Whale, Right Whale data from Sanders et al. [43]). Feeding strategy is specified for sharks and marine mammal only. Alpha diversity is shown as Observed Sequence Variants (SVs, panel **A**) with box plots displaying the median (bold line), 25th–75th percentiles, and whiskers showing a maximum 1.5 × IQR (interquartile range), and beta diversity is shown as a Principal Coordinate Analysis (PCoA) using unweighted UniFrac distances (panel **B**). More information regarding comparative microbiomes can be found in Additional file 1: Table S5

Based on community 16S rRNA gene pools (rarefied to 10,000 sequences per sample), external niches (gill, skin, cloaca) frequently contained over 200 sequence variants (SVs), whereas fecal microbiomes of both *C. carcharias* and *R. typus* (fecal samples were not obtained from *G. cuvier*) contained fewer than 90 sequence variants (range 85–21) (Fig. 5). Fecal microbiomes from *C. carcharias* and *R. typus* were similar in composition. *Photobacterium*, various Clostridia, Campylobacter, and Fusobacteria were present and relatively abundant in both shark species (Additional file 1: Fig. S2), although Fusobacteria were far more abundant in *C. carcharias* compared to *R. typus*. These elasmobranch fecal microbiomes were further analyzed alongside fecal microbiomes from teleost fish [22] and mammals [43]. See Additional file 1: Table S5 for the sample summary. The number of observed SVs was strikingly low in both the apex predator *C. carcharias* and the filter-feeding *R. typus.* In contrast, the number of SVs was low in toothed whales but high in filter-feeding baleen whales (Fig. 6A). Overall, the fecal microbiomes of both shark species clustered most closely with those of carnivorous teleost fishes and toothed whales based on taxonomic composition (unweighted UniFrac, Fig. 6B).

**Fig. 7:**
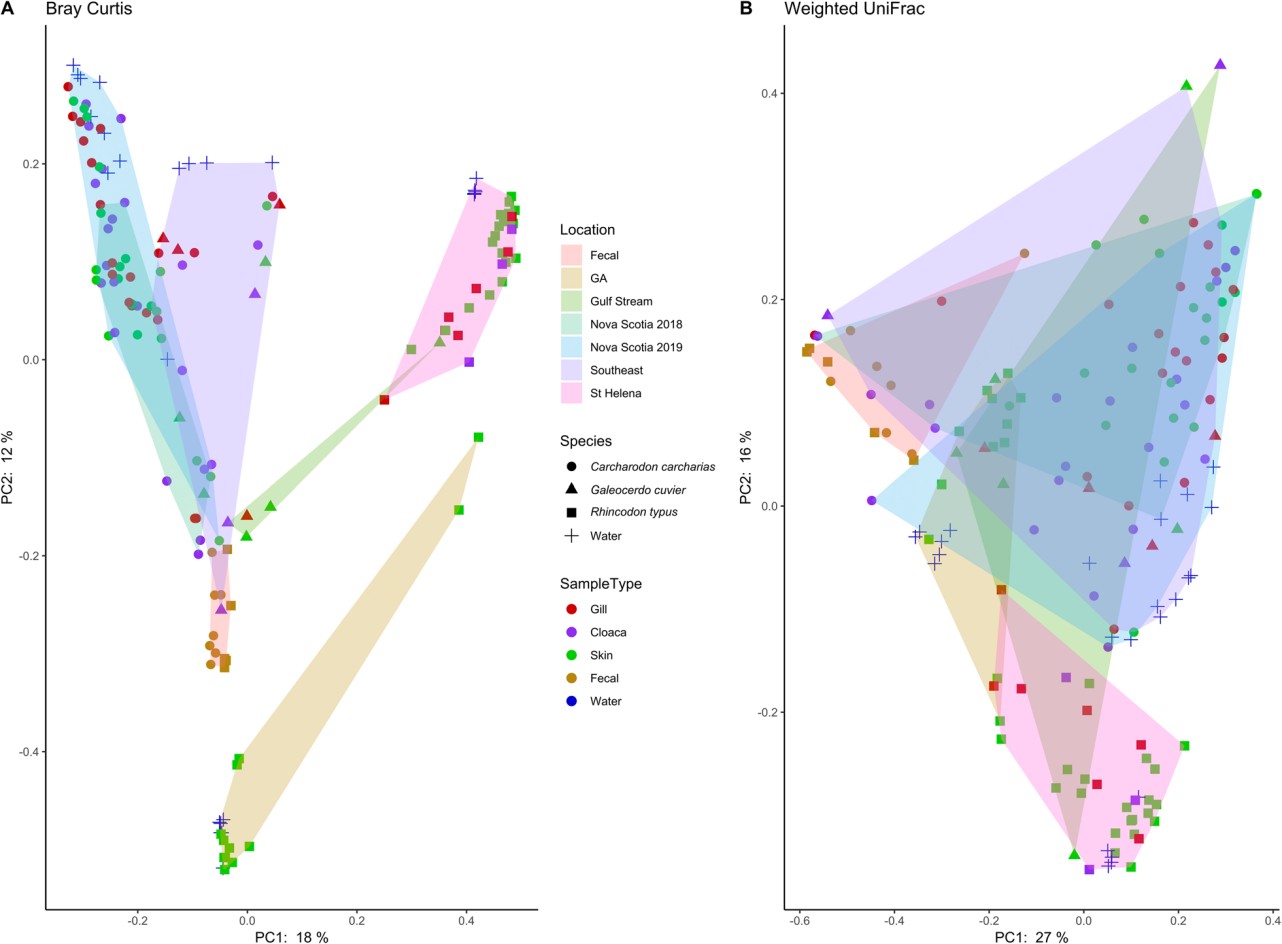
Principal component analysis (PCoA) using Bray–Curtis dissimilarity (panel **A**) and weighted UniFrac (panel **B**) distances for *Carcharodon carcharias*, *Galeocerdo cuvier*, and *Rhincodon typus*. Plots are shaded by the location each shark was located with the exception of fecal samples, as these clustered tighlty together regardless of location. Sample details and numbers are given in Additional file 1: Table S1 and Additional file 1: Table S2

Relatively high alpha diversity was observed in skin microbiomes of managed *R. typus* (Georgia Aquarium), coinciding with elevated diversity in aquarium water microbiomes compared to those of seawater surrounding wild individuals from St. Helena (Additional file 1: Fig. S3). A similar trend was observed for *C. carcharias* samples: decreased cloaca, gill, and skin richness in *C. carcharias* from Nova Scotia was low compared to that of individuals from other locations, coinciding with decreased richness in Nova Scotia seawater. These trends suggest that diversity in external host-associated niches may be influenced by diversity in the surrounding water. An influence of water column microbiomes on those of external body-associated niches was also reflected in analysis of beta diversity (principal component analysis (PCoA) plots of Bray–Curtis dissimilarities), in which samples largely clustered based on geographic location rather than body site (Fig. 7A). Fecal samples were the exception, with fecal communities clustering together regardless of geographic location or even shark species. Separate PCoA analyses for each shark species confirm the pattern of clustering by geography and not body site. In some cases (i.e., collections from the Nova Scotia expeditions), the location-specific clusters included samples collected in two different years (Additional file 1: Fig. S4). In contrast, PCoA plots based upon weighted UniFrac distances showed little separation according to geographic location, body site, or species (Fig. 7B), likely due to broad similarities in community composition at lower taxonomic resolution (Additional file 1: Fig. S5).

Using weighted UniFrac distances, PERMANOVA (Permutational multivariate analysis of variance) was used to compare microbiome taxonomic composition between body sites and the water column from the Nova Scotia 2019 sample set, as that location and year represented the most comprehensive dataset. In pairwise comparisons between body sites, only the cloaca and gill microbiomes differed significantly from one another (Additional file 1: Table S6). However, all body-associated microbiomes differed significantly from those in the surrounding water column.

The Southeast OCEARCH expedition successfully obtained samples from both *C. carcharias* from *G. cuvier* in the same region, enabling us to test for SVs that distinguish the two shark species, independent of confounding effects of geography. Using DESeq2 [44], a total of 139 SVs [41, 45, 53] associated the gills, skin, and cloaca, respectively) varied significantly in proportional abundance between shark species. All of these SVs were classified to the Bacteria domain, with Actinobacteria, Firmicutes, and Fusobacteria-affiliated SVs generally enriched in *G. cuvier* and Verrucomicrobia and Chloroflexi SVs generally enriched in *C. carcharias* (Additional file 1: Table S7).

## Discussion

Feeding behavior may play a relatively minor role in shaping the fecal microbiome of two of the largest migratory animals in the ocean. The fecal microbiomes of the apex predator *C. carcharias* and filter feeder *R. typus* are remarkably similar in taxonomic richness, composition, and (based on limited metagenomic data) broad metabolic category representation. The fecal microbiomes of both shark species are notably taxon-poor, containing roughly one half to one quarter of the SV richness observed in baleen whales, herbivorous teleost fishes, and terrestrial mammalian carnivores and herbivores (Fig. 6) and comprised primarily of bacteria common in fish and other vertebrate microbiomes, including *Photobacterium*, *Fusobacterium, Clostridia* and Campylobacterales. The highly conserved nature of the shark microbiome differs from that of many teleost fish microbiomes, which contain core taxa but also vary widely in composition among host species and individuals of the same species, with much of this variation linked to diet differences [22, 26].

In taxonomic composition, *C. carcharias* and *R. typus* fecal microbiomes are most similar to those of carnivorous teleost fish and apex-predator toothed whales and distinct from those of baleen whales (Fig. 6). The latter result is noteworthy given that whale sharks and baleen whales are filter-feeders and presumably have a similar zooplanktivorous diet. Prior research described the baleen whale microbiome as having similarities to that of terrestrial herbivores, potentially due to shared intestinal morphology (e.g., the presence of a blind-end cecum) and reliance on microbial fermentation in baleen whales and terrestrial herbivores [43]. Our results suggest that the intestinal anatomy/physiology of whale sharks may be sufficiently similar to that of white sharks (and divergent from that of baleen whales) to drive microbiome similarity. Shared factors that may push *C. carcharias* and *R. typus* fecal microbiomes to convergence include the presence of a spiral intestine [38], high urea content in the intestine [45], or the presumed feast and famine nature of shark feeding. Conservation of microbiome structure across phylogenetically related hosts, despite drastic differences in diets, is not unprecedented. For example, the gut microbiome of the herbivorous giant panda (*Ailuropoda melanoleuca*) is more similar to that of carnivorous bears than to phylogenetically distant herbivorous mammals, presumably due to shared gut morphology among bear species [46]. For sharks, we cannot rule out that diet also plays a strong role in microbiome structuring. It is possible that the protein-rich zooplanktivorous diet of whale sharks is sufficiently similar to that of predatory sharks and carnivorous teleosts to drive convergence in white and whale shark microbiomes (and divergence from baleen whale microbiomes). Resolving these hypotheses requires a deeper understanding of shark diets and how digestive organs differ physiologically between predators and filter-feeders. Despite uncertainty about the factors shaping shark microbiomes, our data suggest that fecal microbiome composition—and therefore presumably also the microbiome’s functional contribution to the host—is highly conserved between ecologically distinct shark species.

Representatives of the same microbial species were detected in both *C. carcharias* and *R. typus* fecal microbiomes. These include the ubiquitous fish-associated *Photobacterium damselae*, which was detected in both amplicon and MAG datasets of both sharks (Additional file 1: Fig. S2, Additional file 1: Table S4). The abundance and prevalence of *P. damselae* in these sharks is not surprising. Givens et al. 2015 [26] identified *Photobacterium sp.* and Leigh et al. 2021 [47] identified *P. damselae* as a core member of the gut microbiome of four other shark species, and *P. damselae* has been represented in culture-based studies of the shark gut since 1985 [48]. *Photobacterium damselae* is also common in teleosts [22, 49]. In teleosts, *P. damselae* is a well-known fish (and occasionally human) pathogen [50], and often contains antibiotic resistance genes, as discussed below. Yet it is unlikely that all the sharks examined in this study, and a vast majority of all marine fish, contain *P. damselae* in a disease state. Rather, the ubiquity of this core microbiome member begs a re-examination of its ecological role, which may include functions as a potential symbiont (rather than pathogen) and essential member of the bony and cartilaginous fish gut.

Despite their overall compositional similarity, *C. carcharias* and *R. typus* fecal microbiomes differed in the representation of certain microbial taxa and functional gene categories, raising hypotheses related to diet variation. *Tyzzerella* subgroup 3 (Firmicutes) was substantially enriched in *R. typus*, whereas *Cetobacterium* (Fusobacteria) was enriched in *C. carcharias* (Fig. 2 and Additional file 1: Fig. S2). *Tyzzerella* spp*.* are common commensal gut microbes in vertebrates, including in humans in which *Tyzzerella* has been linked to lower quality diets [51] and maladies such as cardiovascular disease [52], gestational diabetes [53], and irritable bowel syndrome [54]. Although *Tyzzerella* has been detected in marine crustaceans [55, 56] and fish [57, 58], the function of these bacteria and their relationship to diet quality in marine animals, including *R. typus,* is unclear. *Cetobacterium*, as an obligate anaerobe, is also common in vertebrate guts, including in teleost fish where it has been known to produce large quantities of vitamin B-12 [59]. This prior finding raises the possibilities that *Cetobacterium* provides B-12 for *C. carcharias*. Further, the lower abundance of *Cetobacterium* in *R. typus* may indicate that B-12 is relatively more available in the *R. typus* diet. Finally, diet variation may be linked to the varied representation of genes under the COG category "Amino acid metabolism and transport", which were enriched in *C. carcharias* fecal samples compared to those of *R. typus*. Although this comparison is limited by the availability of only one *R. typus* fecal metagenome, a higher abundance of amino acid metabolism in *C. carcharias* may reflect a diet of higher protein content*.* These trends warrant further studies to test the relationship between shark diet and microbial metabolism.

Antibiotic resistance genes were common in *C. carcharias* and *R. typus* fecal microbiomes*,* being detected in 25 of 27 MAGs representing 5 bacterial phyla (Fig. 4), including high quality MAGs classified as *P. damselae*. Antibiotic-resistance genes have been detected in *P. damselae* from fish aquaculture sites [60–62], where regular antibiotic dosing may select for both the retention and transfer of resistance among enteric microbes [63]. In wild animals, antibiotic resistance genes tend to be more common with increased exposure to anthropogenic impacts [64]. For migratory sharks, we have limited data on the baseline frequency of antibiotic-resistance in microbiomes, nor do we know enough about migration patterns to assess the relative exposure of sharks to anthropogenic impacts, potentially including exposure to taxa such as *P. damselae* whose resistance profile could be shaped by human activity. Despite this uncertainty, it is most likely that the high representation of resistance genes in *C. carcharias* and *R. typus* fecal microbiomes is driven by natural, antagonistic interactions between microbes. Prior work has shown that bacteria from elasmobranch skin also produce an array of antimicrobial compounds [12]. Intriguingly, sharks rarely suffer wound infections [11, 65, 66], and it is possible that microbial warfare by the resident microbiome plays a role in disease prevention. Collectively, the available data suggest that microbial warfare is common in the shark microbiome and that these communities may be repositories for both antibiotics and antibiotic-resistance. Metagenome and culture-based studies that compare microbiome niches on the shark body, as well as individuals in wild versus managed care environments, will help better resolve the nature and role of microbe-microbe interactions in sharks.

Unlike the fecal microbiome, external (skin, gill, cloaca) microbiomes on sharks in this study are strongly influenced by microbiomes of the surrounding seawater (Figs. 5, 6). A strong environmental influence on microbiome structuring has been documented in other marine animals, including the common thresher shark (*Alopias vulpinus* [24]), the blacktip reef shark (*Carcharhinus melanopterus* [25]), orcas (*Orca orcinus* [67]), and humpback whales (*Megaptera novaeangliae* [68]). However, this influence is less pronounced in certain teleost fishes [23, 69], although still important [21, 69]. A relative lack of host-mediated regulation of external microbiomes in sharks may be due to the unique structure of the shark skin, consisting of rough dermal denticles that are absent in teleost fishes. The surface of the shark skin may also contain less mucus, thereby limiting the development and growth of a resident biofilms. Nonetheless, the lack of significant microbiome variation between *C. carcharias* and *G. cuvier* from the same location was surprising, notably as *G. cuvier* is known to contain more mucus on its skin. Additionally, prior work on other elasmobranch species shows that skin microbiomes tend to follow a pattern phylosymbiosis, being distinct among shark hosts [23]. The similarity between *C. carcharias* and *G. cuvier* skin, gill, and cloaca microbiomes may be driven by lifestyle similarity between these two apex predators, a particularly strong environmental effect during our sampling, and potentially convergent microbiome evolution, which can confound phylosymbiosis signals.

## Conclusions

Our knowledge of shark-microbe interactions and their role in shark health and ecology is increasing, but remains sparse for large pelagic sharks. For such species, the expertise and resources required to find, capture, and sample wild individuals remains a primary challenge. The current study, made possible by a large collaborative effort uniting marine biologists, microbiologists, and specialists in large animal handling, establishes critical baseline data and hypotheses to guide future investigations. The fecal microbiomes of the large pelagic sharks *C. carcharias* and *R. typus* are simple and conserved, despite host differences in feeding mode and apparent similarities in trophic niche between *R. typus* and baleen whales. Both species are enriched in core microbiome members including *Photobacterium*, whose abundance and ubiquity in the shark gut warrants attention to identify its functional contributions and potential strain-variation among hosts (comparisons of MAGs between hosts was hindered here by the low quality of *P. damselae* MAGs in *R. typus;* Additional file 1: Table S4)*.* Future studies should also target other dominant taxa such as *Cetobacterium* and *Tyzzerlla,* as well as expand metagenomic comparisons. The latter will allow testing of the natural product potential of shark microbes and the role of social interactions among microbiome members (e.g., pathogen suppression). If coupled to data on shark diet, intestinal physiology, and migration patterns, future microbiome studies can help resolve the contributions of diet versus physiology versus environmental factors in shaping microbiome function, enabling predictions of how shark health and ecology may change with anthropogenic stress to shark populations, or care under managed conditions.

## Materials and methods

### Experimental design

Additional file 1: Table S1 lists all samples. Tiger shark (*Galeocerdo cuvier*) and white shark (*Carcharodon carcharias*) samples were collected in collaboration with OCEARCH over four expeditions: 2018 Gulf Stream, 2018 Nova Scotia, 2019 Southeast (Florida, Georgia, South Carolina), 2019 Nova Scotia (Additional file 1: Table S2 lists metadata and shark names for all OCEARCH samples). Science Briefs for each expedition can be found at https://www.ocearch.org/. Microbiome samples were collected from the dorsal skin, cloaca, gill, or feces from individuals of *Galeocerdo cuvier* (tiger shark; n = 7 individuals) and *Carcharodon Carcharias* (white shark; n = 22). Not all sample types were collected from all individuals (Additional file 1: Table S1). Shark capture and sampling followed OCEARCH procedures. Briefly, animals were caught with drumlines from a tender boat and then guided to a submerged platform of the *M/V OCEARCH* vessel. The platform was then raised, the animal restrained, and ventilated with equipment/hoses to provide a continuous flow of fresh seawater over the gills. The science team of researchers and veterinarians then sampled the animal for ~ 15 min prior to release. Skin, cloaca, and gill samples were collected using a sterile swab brushed approximately 10–15 times over the tissue surface. Swabs were then placed in a vial containing an RNA/DNA stabilizing buffer (25 mM sodium citrate, 10 mM EDTA, 5.3 M ammonium sulfate, pH 5.2) and frozen until extraction. Fecal samples were rare as only seven *C. carcharias* individuals had obtainable fecal matter; fecal samples were not obtained from *G. cuvier*. *Carcharodon carcharias* fecal samples were collected directly from the spiral intestine with a rubber catheter and syringe, transferred to a cryovial, stored frozen within 20 min of collection, and kept frozen until DNA extraction.

Samples of *Rhincodon typus* (whale shark) microbiomes were collected via freediving from four locations, representing a total of 37 individuals (Additional file 1: Table S1). The dorsal pit (beneath/posterior to the first dorsal fin) of four managed care whale sharks (*Rhincodon typus*) from Georgia Aquarium were sampled six different times over a period of five months, and multiple body sites were sampled for 29 wild whale sharks from the south Atlantic island of St. Helena; in addition, one fecal sample was collected. Additional whale shark fecal samples were obtained from three wild individuals in Tanzania and one from the Maldives. Samples of dorsal skin/pit, cloaca, and gill microbiomes were collected by gently brushing the surfaces with a sterile swab and preserved in an RNA/DNA stabilizing buffer as described above. Fecal samples from *R. typus* were collected from the water column directly after defecation events that occurred while freediving. Feces were collected by hand using nets or containers and frozen as quickly as possible. This research was approved by the Georgia Institute of Technology Institutional Animal Care and Use Committee (IACUC) under protocols A100161 and A100122.

Surface seawater from the sites of wild animal sampling was collected via two sterile 60 ml syringes. Seawater microbiomes were then concentrated by filtering the 120 ml of water through a 0.2 µm Isopore membrane filter (Millipore). To collect Georgia Aquarium seawater microbiomes, 2 L of exhibit water were passed through a 0.2 µm Sterivex filter (see [70]). Filters were immersed in RNA/DNA stabilizing buffer and frozen until extraction.

### DNA extraction, library preparation, and sequencing

Total DNA was extracted from all samples using the Qiagen PowerSoil DNAextraction kit. Swabs, Isopore membrane filters, or 200 μl of fecal content were placed directly into PowerBead tubes, and extracted following the manufacturers’ instructions, with two exceptions; samples were heated to 55 °C for five minutes before the bead beating step and before the final elution. Fecal samples were extracted in duplicate. Microbial community composition was assessed by Illumina sequencing of the V4 region of the 16S rRNA gene, as described by the Earth Microbiome Project [71]. A nested PCR approach was necessary for successful amplicon generation for *Carcharodon carcharias* and *Galeocerdo cuvier*, first targeting the full 16S rRNA gene, then applying a second PCR targeting the V4 region of the 16S rRNA gene. The first PCR included 5 μl DNA template (with the exception of fecal samples, which included 2 ul DNA template), 12.5 μl Promega GoTaq Hot Start Green Master Mix, 0.5 μl each 27F forward primer and 1492R reverse primer (total concentration, 0.2 μM), and 0.25 μl bovine serum albumin (BSA) (20 mg/ml; ThermoScientific). Final reaction volumes were brought up to 25 μl. PCR conditions were an initial 3-min denaturation at 94 °C, followed by 30 cycles of denaturation at 94 °C (60 s), primer annealing at 55 °C (120 s), and extension at 72 °C (90 s) and then a final extension at 72 °C for 10 min. After the initial PCR, a second nested PCR was performed using 0.25 μl template from the first PCR, and 0.25 μl each F515 forward primer and R806 reverse primer [72], both with appended barcodes and Illumina-specific adapters [73]. All other PCR conditions were described as above. A nested PCR approach was not necessary for *R. typus* samples, where 5 μl of DNA template was used in PCR conditions as described above. All DNA extractions were amplified in triplicate, each replicate with their own barcode combination. Blanks (negative controls) were carried out through extraction, PCR and sequencing for all protocol variants. Amplicon libraries were purified with Diffinity RapidTip PCR purification tips (Diffinity Genomics, NY) and pooled. 16S amplicon libraries were sequenced on an Illumina MiSeq machine using V2 chemistry with 500 cycles (250 × 250 bp) and 5% PhiX. Metagenome libraries from each fecal extraction were constructed using the Nextera XT DNA sample prep kit (Illumina) per the manufacturer’s instructions. The resulting libraries were verified on the 2100 Bioanalyzer (Agilent) and sequenced using V3 chemistry with 600 cycles (300 × 300 bp) and 5% PhiX. One white shark fecal sample (SE20193) was chosen for long-read metagenome scaffolding using Nanopore Technologies' Ligation Sequencing Kit and the MinION Mk1B. Extraction blanks were performed and carried out through PCR and sequencing. All sequence data generated in this study have been deposited in the NCBI Sequence Read Archive under BioProject ID numbers PRJNA648940 and PRJNA649531.

### 16S rRNA gene amplicon analysis

Raw reads were imported into QIIME2, and sequences were quality filtered, merged, and checked for chimeras using the DADA2 pipeline with the parameters -p-trim-left-f 100 –p-trim-left-r 100 –p-trunc-len-f 140 –p-trunc-len-r 140 [74]. Quality filtered reads were assigned taxonomy using the SILVA pre-trained classifier (silva-132-99-515-806-nb-classifier), and all chloroplast, mitochondria, and "unassigned" reads at the domain level were removed. A total of 9,539,619 total sequences were retained after quality control, ranging from 1818 to 436,939 sequences per sample, and comprising 11,047 unique sequence variants (SVs) across 161 samples.

To control for PCR bias between replicates, we applied a quality control procedure based on weighted UniFrac distances. Distances were calculated for all replicates, and distances between replicates were plotted in a standard boxplot. Outlier replicates (those with distances in the fourth quartile, > 0.33) were then removed (Additional file 1: Fig. S6). This quality control process also identified all negative controls for removal from the dataset, further validating the method. Replicates that passed this quality control were combined for further processing. Counts of observed SVs, Chao1 estimates of SV richness (alpha diversity), Bray–Curtis dissimilarity values, and weighted UniFrac distances (beta diversity) were calculated through QIIME2, and .qza files imported into R using the package QIIME2R for figure production. Only the SE2019 expedition yielded a sample set representing two species (*Carcharodon carcharias* and *Galeocerdo cuvier*)*,* and was therefore isolated to test the effects of a shared environment versus host identity on microbiome composition. PERMANOVA was used to test for variation in community composition among sample types (gill, skin, cloaca, feces) in QIIME2. Nonrarefied count data were imported into R using the packages Qiime2R and Phyloseq [75], and DESeq2 [44] was used to detect SVs that differed significantly in proportional abundance among sample groups. Data from the NS2019 expedition were also evaluated independently to test for significant differences in microbiome composition between body sites and the water column, as this study site had the most water samples collected (n = 8). Two previous studies were selected for comparison to further elucidate the roles of host identity vs diet in the shark fecal microbiome- one with fecal microbiomes from a diverse set of marine teleost fishes spanning disparate diets [22], and one examining the fecal microbiomes of a diverse set of mammals [43], including toothed and baleen whales, which have similar diets to white and whale sharks, respectively. Raw data from these two studies [43 and 22] were processed with the same QIIME2 pipeline described above, except that only forward reads were used.

### Metagenomic analysis

Demultiplexed reads were trimmed using Trim Galore, with the criteria of a minimum length of 100 bp and a Phred score of 25. Long-read MinION data produced from the SE20193 fecal sample were trimmed in the same manner except with a minimum length of 1000 bp. For each fecal sample, the quality trimmed forward and reverse sequences were assembled into contigs using SPAdes 3.13.0 using the metaspade.py script [76]. Contigs of > 5000 bp were binned into metagenome-assembled genomes (MAGs) using MaxBin2.0 [77]. MAG completeness, contamination, average nucleotide identity (ANI), and taxonomic affiliation were determined using the program MiGA [78]. For the MAGs identified as *Photobacterium*, a phylogenetic tree was constructed along with 26 closely related genomes on the basis of 91 concatenated single-copy marker genes present in all MAGs and identified by MiGA using Hidden Markov Models (HMMs) via HMMER3. Marker genes were aligned using clustalW and then concatenated in MEGAX. The resulting alignment was manually verified and used to generate a maximum likelihood phylogeny inferred with the Dayhoff substitution model and iterated 500 times for bootstrapping [79, 80].

For comparative analysis, metagenomic reads from all *C. carcharias* and *R. typus* fecal samples were co-assembled using MEGAHIT v. 1.2.9 [81]. The co-assembly was imported into Anvi'o 6.2 [41] and binned using Maxbin 2.0 [76]. Individual metagenomes were then mapped back to co-assembly bins using Bowtie2 2.3.5 [82]. Genes were annotated using the Clusters of Orthologous Groups (COGs) database as in [83], and the relative abundance of each COG category was calculated for each metagenome. Bins across all samples were then manually refined and visualized in the Anvi'o interactive interface.

## Supplementary Information

**Additional file 1.** Figures S1–S6 and Tables S1–S7 reporting sample identity and collection information, microbiome diversity statistics, and additional metagenomic results.

## Data Availability

All sequence data generated in this study have been deposited in the NCBI Sequence Read Archive under BioProject ID numbers PRJNA648940 and PRJNA649531.

## Abbreviations

ANI: Average nucleotide similarity
BSA: Bovine serum albumin
COGs: Clusters of Orthologous Groups
IACUC: Institutional Animal Care and Use Committee
MAG: Metagenome assembled genome
PCoA: Principal component analysis
PCR: Polymerase chain reaction
SVs: Sequence variants

## References

[1] Heithaus MR, Frid A, Wirsing AJ, Worm B (2008). Predicting ecological consequences of marine top predator declines. Trends Ecol Evol.

[2] Ruppert JL, Travers MJ, Smith LL, Fortin MJ, Meekan MG (2013). Caught in the middle: combined impacts of shark removal and coral loss on the fish communities of coral reefs. PLoS ONE.

[3] Cisneros-Montemayor AM, Barnes-Mauthe M, Al-Abdulrazzak D, Navarro-Holm E, Sumaila UR (2013). Global economic value of shark ecotourism: implications for conservation. Oryx.

[4] Dent F, Clarke S (2015). State of the global market for shark products. FAO Fish Aquac.

[5] Criscitiello MF (2014). What the shark immune system can and cannot provide for the expanding design landscape of immunotherapy. Expert Opin Drug Discov.

[6] Marra NJ, Richards VP, Early A, Bogdanowicz SM, Bitar PDP, Stanhope MJ, Shivji MS (2017). Comparative transcriptomics of elasmobranchs and teleosts highlight important processes in adaptive immunity and regional endothermy. BMC Genom.

[7] Rasch LJ, Martin KJ, Cooper RL, Metscher BD, Underwood CJ, Fraser GJ (2016). An ancient dental gene set governs development and continuous regeneration of teeth in sharks. Dev Biol.

[8] Moore KS, Wehrli S, Roder H, Rogers M, Forrest JN, McCrimmon D, Zasloff M (1993). Squalamine: an aminosterol antibiotic from the shark. PNAS.

[9] Mann EE, Manna D, Mettetal MR, May RM, Dannemiller EM, Chung KK, Brennan AB, Reddy ST (2014). Surface micropattern limits bacterial contamination. Antimicrob Resist Infect Control.

[10] Towner A, Smale MJ, Jewell O. Boat strike wound healing in *Carcharodon carcharias.* In: Global perspectives on the biology and life history of the white shark; 2012. p. 77–84.

[11] Chin A, Mourier J, Rummer JL (2015). Blacktip reef sharks (*Carcharhinus melanopterus*) show high capacity for wound healing and recovery following injury. Conserv Physiol.

[12] Ritchie KB, Schwarz M, Mueller J, Lapacek VA, Merselis D, Walsh CJ, Luer CA (2017). Survey of antibiotic-producing bacteria associated with the epidermal mucus layers of rays and skates. Front Microbiol.

[13] Dulvy NK, Fowler SL, Musick JA, Cavanagh RD, Kyne PM, Harrison LR, Carlson JK, Davidson LN, Fordham SV, Francis MP, Pollock CM (2014). Extinction risk and conservation of the world’s sharks and rays. Elife.

[14] MacNeil MA, Chapman DD, Heupel M, Simpfendorfer CA, Heithaus M, Meekan M, Harvey E, Goetze J, Kiszka J, Bond ME, Currey-Randall LM (2020). Global status and conservation potential of reef sharks. Nature.

[15] Hooper LV, Littman DR, Macpherson AJ (2012). Interactions between the microbiota and the immune system. Science.

[16] Hacquard S, Garrido-Oter R, González A, Spaepen S, Ackermann G, Lebeis S, McHardy AC, Dangl JL, Knight R, Ley R, Schulze-Lefert P (2015). Microbiota and host nutrition across plant and animal kingdoms. Cell Host Microbe.

[17] Cantley AM, Clardy J (2015). Animals in a bacterial world: opportunities for chemical ecology. Nat Prod Rep.

[18] Gilbert JA, Quinn RA, Debelius J, Xu ZZ, Morton J, Garg N, Jansson JK, Dorrestein PC, Knight R (2016). Microbiome-wide association studies link dynamic microbial consortia to disease. Nature.

[19] McFall-Ngai M, Hadfield MG, Bosch TCG, Carey HV, Domazet-Lošo T, Douglas AE, Dubilier N, Eberl G, Fukami T, Gilbert SF, Hentschel U, King N, Kjelleberg S, Knoll AH, Kremer N, Mazmanian SK, Metcalf JL, Nealson K, Pierce NE, Rawls JF, Reid A, Ruby EG, Rumpho M, Sanders JG, Tautz D, Wernegreen JJ (2013). Animals in a bacterial world, a new imperative for the life sciences. PNAS.

[20] Leftwich PT, Clarke NVE, Hutchings MI, Chapman T (2017). Gut microbiomes and reproductive isolation in *Drosophila*. PNAS.

[21] Chiarello M, Auguet J-C, Bettarel Y, Bouvier C, Claverie T, Graham NAJ, Rieuvilleneuve F, Sucré E, Bouvier T, Villéger S (2018). Skin microbiome of coral reef fish is highly variable and driven by host phylogeny and diet. Microbiome.

[22] Pratte ZA, Besson M, Hollman RD, Stewart FJ (2018). The gills of reef fish support a distinct microbiome influenced by host-specific factors. AEM.

[23] Doane MP, Morris MM, Papudeshi B, Allen L, Pande D, Haggerty JM, Johri S, Turnlund AC, Peterson M, Kacev D, Nosal A, Ramirez D, Hovel K, Ledbetter J, Alker A, Avalos J, Baker K, Bhide S, Billings E, Byrum S, Clemens M, Demery AJ, Lima LFO, Gomez O, Gutierrez O, Hinton S, Kieu D, Kim A, Loaiza R, Martinez A, McGhee J, Nguyen K, Parlan S, Pham A, Price-Waldman R, Edwards RA, Dinsdale EA (2020). The skin microbiome of elasmobranchs follows phylosymbiosis, but in teleost fishes, the microbiomes converge. Microbiome.

[24] Doane MP, Haggerty JM, Kacev D, Papudeshi B, Dinsdale EA (2017). The skin microbiome of the common thresher shark (*Alopias vulpinus*) has low taxonomic and gene function β-diversity. Environ Microbiol Rep.

[25] Pogoreutz C, Gore MA, Perna G, Millar C, Nestler R, Ormond RF, Clarke CR, Voolstra CR (2019). Similar bacterial communities on healthy and injured skin of black tip reef sharks. Anim Microbiome.

[26] Givens C, Ransom B, Bano N, Hollibaugh T (2015). Comparison of the gut microbiomes of 12 bony fish and 3 shark species. Mar Ecol Prog Ser.

[27] Zmora N, Suez J, Elinav E (2019). You are what you eat: diet, health and the gut microbiota. Nat Rev Gastroenterol Hepatol.

[28] Dicken ML (2008). First observations of young of the year and juvenile great white sharks (*Carcharodon carcharias*) scavenging from a whale carcass. Mar Freshw Res.

[29] Domeier ML (2009). Experimental scavenging preference for the adult white shark, *Carcharodon carcharias*. Calif Fish Game.

[30] Klimley AP, Le Boeuf BJ, Cantara KM, Richert JE, Davis SF, Van Sommeran S, Kelly JT (2001). The hunting strategy of white sharks (*Carcharodon carcharias*) near a seal colony. Mar Biol.

[31] Heithaus MR, Dill LM, Marshall GJ, Buhleier BM (2002). Habitat use and foraging behavior of tiger sharks (*Galeocerdo cuvier*) in a seagrass ecosystem. Mar Biol.

[32] Semmens JM, Payne NL, Huveneers C, Sims DW, Bruce BD (2013). Feeding requirements of white sharks may be higher than originally thought. Sci Rep.

[33] Motta PJ, Wilga CD, Tricas TC, Gruber SH (2001). Advances in the study of feeding behaviors, mechanisms, and mechanics of sharks. The behavior and sensory biology of elasmobranch fishes: an anthology in memory of Donald Richard Nelson.

[34] Rowat D, Brooks KS (2012). A review of the biology, fisheries and conservation of the whale shark *Rhincodon typus*. J Fish Biol.

[35] Rohner CA, Couturier LIE, Richardson AJ, Pierce SJ, Prebble CEM, Gibbons MJ, Nichols PD (2013). Diet of whale sharks *Rhincodon typus* inferred from stomach content and signature fatty acid analyses. Mar Ecol Prog Ser.

[36] Wyatt ASJ, Matsumoto R, Chikaraishi Y, Miyairi Y, Yokoyama Y, Sato K, Ohkouchi N, Nagata T (2019). Enhancing insights into foraging specialization in the world's largest fish using a multi-tissue, multi-isotope approach. Ecol Monogr.

[37] Motta PJ, Maslanka M, Hueter RE, Davis RL, De la Parra R, Mulvany SL, Habegger ML, Strother JA, Mara KR, Gardiner JM, Tyminski JP (2010). Feeding anatomy, filter-feeding rate, and diet of whale sharks *Rhincodon typus* during surface ram filter feeding off the Yucatan Peninsula, Mexico. Zoology.

[38] Leigh SC, Papastamatiou Y, German DP (2017). The nutritional physiology of sharks. Rev Fish Biol Fish.

[39] Domeier ML, Nasby-Lucas N. Sex-specific migration patterns and sexual segregation of adult white sharks, *Carcharodon carcharias*, in the Northeastern Pacific. In: *Global perspectives on the biology and life history of the white shark*. Boca Raton: CRC Press; 2012. P.133–46.

[40] Bastien G, Barkley A, Chappus J, Heath V, Popov S, Smith R, Tran T, Currier S, Fernandez DC, Okpara P, Owen V (2020). Inconspicuous, recovering, or northward shift: status and management of the white shark (*Carcharodon carcharias*) in Atlantic Canada. Can J Fish Aquat Sci.

[41] Eren AM, Esen ÖC, Quince C, Vineis JH, Morrison HG, Sogin ML, Delmont TO (2015). Anvi’o: an advanced analysis and visualization platform for ‘omics data. PeerJ.

[42] Balado M, Benzekri H, Labella AM, Claros MG, Manchado M, Borrego JJ, Osorio CR, Lemos ML (2017). Genomic analysis of the marine fish pathogen *Photobacterium damselae* subsp. piscicida: insertion sequences proliferation is associated with chromosomal reorganisations and rampant gene decay. Infect Genet Evol.

[43] Sanders JG, Beichman AC, Roman J, Scott JJ, Emerson D, McCarthy JJ, Girguis PR (2015). Baleen whales host a unique gut microbiome with similarities to both carnivores and herbivores. Nat Commun.

[44] Love MI, Huber W, Anders S (2014). Moderated estimation of fold change and dispersion for RNA-seq data with DESeq2. Genome Biol.

[45] Wood CM, Kajimura M, Bucking C, Walsh PJ (2007). Osmoregulation, ionoregulation and acid–base regulation by the gastrointestinal tract after feeding in the elasmobranch (*Squalus acanthias*). J Exp Biol.

[46] Ley RE, Hamady M, Lozupone C, Turnbaugh PJ, Ramey RR, Bircher JS, Schlegel ML, Tucker TA, Schrenzel MD, Knight R, Gordon JI (2008). Evolution of mammals and their gut microbes. Science.

[47] Leigh SC, Papastamatiou YP, German DP (2021). Gut microbial diversity and digestive function of an omnivorous shark. Mar Biol.

[48] Grimes DJ, Brayton P, Colwell RR, Gruber SH (1985). Vibrios as autochthonous flora of neritic sharks. Syst Appl Microbiol.

[49] Parris DJ, Morgan MM, Stewart FJ (2019). Feeding rapidly alters microbiome composition and gene transcription in the clownfish gut. Appl Environ Microbiol.

[50] Rivas A, Lemos M, Osorio C (2013). *Photobacterium damselae* subsp. damselae, a bacterium pathogenic for marine animals and humans. Front Microbiol.

[51] Liu Y, Ajami NJ, El-Serag HB, Hair C, Graham DY, White DL, Chen L, Wang Z, Plew S, Kramer J, Cole R (2019). Dietary quality and the colonic mucosa–associated gut microbiome in humans. Am J Clin Nutr.

[52] Ascher S, Reinhardt C (2018). The gut microbiota: an emerging risk factor for cardiovascular and cerebrovascular disease. Eur J Immunol.

[53] Ma S, You Y, Huang L, Long S, Zhang J, Guo C, Zhang N, Wu X, Xiao Y, Tan H (2020). Alterations in gut microbiota of gestational diabetes patients during the first trimester of pregnancy. Front Cell Infect Microbiol.

[54] Zhuang X, Tian Z, Li L, Zeng Z, Chen M, Xiong L (2018). Fecal microbiota alterations associated with diarrhea-predominant irritable bowel syndrome. Front Microbiol.

[55] Shui Y, Guan ZB, Liu GF, Fan L (2020). Gut microbiota of red swamp crayfish *Procambarus clarkii* in integrated crayfish-rice cultivation model. AMB Expr.

[56] Sun Y, Han W, Liu J, Huang X, Zhou W, Zhang J, Cheng Y (2020). Bacterial community compositions of crab intestine, surrounding water, and sediment in two different feeding modes of *Eriocheir sinensis*. Aquac Rep.

[57] Cerqueda-García D, Améndola-Pimenta M, Zamora-Briseño JA, González-Penagos CE, Árcega-Cabrera F, Ceja-Moreno V, Rodríguez-Canul R (2020). Effects of chronic exposure to water accommodated fraction (WAF) of light crude oil on gut microbiota composition of the lined sole (*Achirus lineatus*). Mar Environ Res.

[58] Hassenrück C, Reinwald H, Kunzmann A, Tiedemann I, Gärdes A (2021). Effects of thermal stress on the gut microbiome of Juvenile Milkfish (*Chanos chanos*). Microorganisms.

[59] Tsuchiya C, Sakata T, Sugita H (2008). Novel ecological niche of *Cetobacterium somerae*, an anaerobic bacterium in the intestinal tracts of freshwater fish. Lett Appl Microbiol.

[60] Morii H, Bharadwaj MS, Eto N (2004). Cloning and nucleotide sequence analysis of the ampicillin resistance gene on a conjugative R plasmid from the fish pathogen *Photobacterium damselae* subsp. *piscicida*. J Aquat Anim Health.

[61] Kim MJ, Hirono I, Kurokawa K, Maki T, Hawke J, Kondo H, Santos MD, Aoki T (2008). Complete DNA sequence and analysis of the transferable multiple-drug resistance plasmids (R plasmids) from *Photobacterium damselae* subsp. *piscicida*; isolates collected in Japan and the United States. Antimicrob Agents Chemother.

[62] Nonaka L, Maruyama F, Miyamoto M, Miyakoshi M, Kurokawa K, Masuda M (2012). Novel conjugative transferable multiple drug resistance plasmid pAQU1 from *Photobacterium damselae* subsp. *damselae* isolated from marine aquaculture environment. Microbes Environ.

[63] Cabello FC (2006). Heavy use of prophylactic antibiotics in aquaculture: a growing problem for human and animal health and for the environment. Environ Microbiol.

[64] Allen HK, Donato J, Wang HH, Cloud-Hansen KA, Davies J, Handelsman J (2010). Call of the wild: antibiotic resistance genes in natural environments. Nat Rev Microbiol.

[65] Ostrander GK, Cheng KC, Wolf JC, Wolfe MJ (2004). Shark cartilage, cancer and the growing threat of pseudoscience. Cancer Res.

[66] Luer CA, Walsh CJ (2018). Potential human health applications from marine biomedical research with elasmobranch fishes. Fishes.

[67] Hooper R, Brealey JC, van der Valk T, Alberdi A, Durban JW, Fearnbach H, Robertson KM, Baird RW, Bradley Hanson M, Wade P, Gilbert MTP, Morin PA, Wolf JBW, Foote AD, Guschanski K (2019). Host-derived population genomics data provides insights into bacterial and diatom composition of the killer whale skin. Mol Ecol.

[68] Apprill A, Robbins J, Eren AM, Pack AA, Reveillaud J, Mattila D, Moore M, Niemeyer M, Moore KMT, Mincer TJ (2014). Humpback whale populations share a core skin bacterial community: towards a health index for marine mammals?. PLoS ONE.

[69] Krotman Y, Yergaliyev TM, Alexander Shani R, Avrahami Y, Szitenberg A (2020). Dissecting the factors shaping fish skin microbiomes in a heterogeneous inland water system. Microbiome.

[70] Patin NV, Pratte ZA, Regensburger M, Hall E, Gilde K, Dove ADM, Stewart FJ (2019). Microbiome dynamics in a large artificial seawater aquarium. AEM.

[71] Thompson LR, Sanders JG, McDonald D, Amir A, Ladau J, Locey KJ, Prill RJ, Tripathi A, Gibbons SM, Ackermann G, Navas-Molina JA, Janssen S, Kopylova E, Vázquez-Baeza Y, González A, Morton JT, Mirarab S, Zech XuZ, Jiang L, Haroon MF, Kanbar J, Zhu Q, Jin Song S, Kosciolek T, Bokulich NA, Lefler J, Brislawn CJ, Humphrey G, Owens SM, Hampton-Marcell J, Berg-Lyons D, McKenzie V, Fierer N, Fuhrman JA, Clauset A, Stevens RL, Shade A, Pollard KS, Goodwin KD, Jansson JK, Gilbert JA, Knight R, Consortium TEMP (2017). A communal catalogue reveals Earth’s multiscale microbial diversity. Nature.

[72] Caporaso JG, Lauber CL, Walters WA, Berg-Lyons D, Lozupone CA, Turnbaugh PJ, Fierer N, Knight R (2011). Global patterns of 16S rRNA diversity at a depth of millions of sequences per sample. PNAS.

[73] Kozich JJ, Westcott SL, Baxter NT, Highlander SK, Schloss PD (2013). Development of a dual-index sequencing strategy and curation pipeline for analyzing amplicon sequence data on the MiSeq Illumina sequencing platform. AEM.

[74] Callahan BJ, McMurdie PJ, Rosen MJ, Han AW, Johnson AJA, Holmes SP (2016). DADA2: High-resolution sample inference from Illumina amplicon data. Nat Methods.

[75] McMurdie PJ, Holmes S (2013). phyloseq: an R package for reproducible interactive analysis and graphics of microbiome census data. PLoS ONE.

[76] Bankevich A, Nurk S, Antipov D, Gurevich AA, Dvorkin M, Kulikov AS, Lesin VM, Nikolenko SI, Pham S, Prjibelski AD, Pyshkin AV, Sirotkin AV, Vyahhi N, Tesler G, Alekseyev MA, Pevzner PA (2012). SPAdes: a new genome assembly algorithm and its applications to single-cell sequencing. J Comput Biol.

[77] Wu YW, Simmons BA, Singer SW (2015). MaxBin 2/0: an automated binning algorithm to recover genomes from multiple metagenomic datasets. Bioinformatics.

[78] Rodriguez RLM, Gunturu S, Harvey WT, Rosselló-Mora R, Tiedje JM, Cole JR, Konstantinidis KT (2018). The Microbial Genomes Atlas (MiGA) webserver: taxonomic and gene diversity analysis of Archaea and Bacteria at the whole genome level. Nucleic Acids Res.

[79] Dayhoff M, Schwartz R, Orcutt B (1978). A model of evolutionary change in proteins. Atlas of protein sequence and structure.

[80] Kumar S, Stecher G, Li M, Knyaz C, Tamura K (2018). MEGA X: Molecular evolutionary genetics analysis across computing platforms. Mol Biol Evol.

[81] Li D, Liu CM, Luo R, Sadakane K, Lam TW (2015). MEGAHIT: an ultra-fast single-node solution for large and complex metagenomics assembly via succinct de Bruijn graph. Bioinformatics.

[82] Langmead B, Salzberg SL (2012). Fast gapped-read alignment with Bowtie 2. Nat Methods.

[83] Galperin MY, Makarova KS, Wolf YI, Koonin EV (2015). Expanded microbial genome coverage and improved protein family annotation in the COG database. Nucleic Acids Res.

