# Supplementary material for: Microbiome structure in large pelagic sharks with distinct feeding ecologies
Source: Anim Microbiome. 2022 Mar 4;4:17. doi: 10.1186/s42523-022-00168-x (PMC8895868; doi:10.1186/s42523-022-00168-x)
Supplement: Supplementary file 1 — Additional file 1. Figures S1–S6 and Tables S1–S7 reporting sample identity and collection information, microbiome diversity statistics, and additional metagenomic results. [file 42523_2022_168_MOESM1_ESM.pdf]

Supplementary Materials for

Microbiome structure in large pelagic sharks with distinct feeding ecologies

Zoe A. Pratte*, Cameron Perry, Alistair D.M. Dove, Lisa A. Hoopes, Kim B. Ritchie, Robert E. Hueter, Chris Fischer, Alisa Newton, and Frank J. Stewart

*Corresponding author. Email: zoe.pratte@montana.edu

This PDF file includes: Supplementary Figures 1 - 5 and Supplementary Tables 1 - 7.

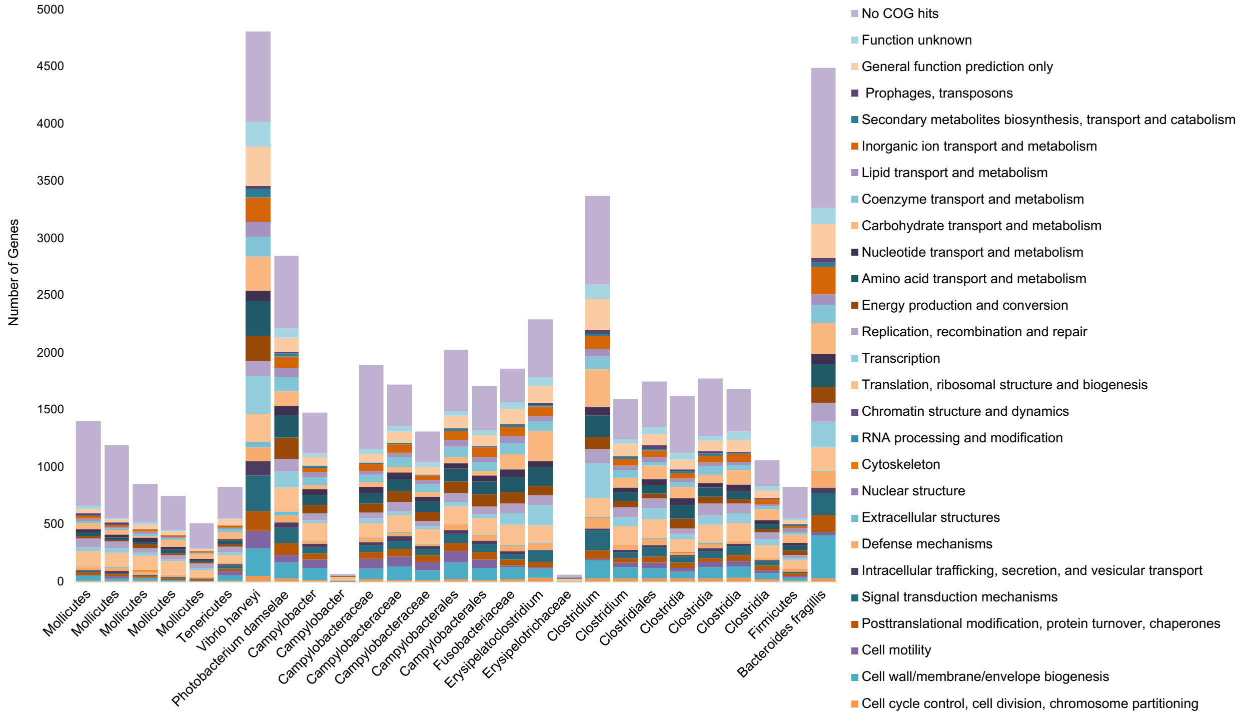

Supplemental Figure 1. Clusters of Orthologous Groups (COG) gene calls of Anvi'o produced bins. The "Defense mechanisms" category is further broken down in Figure 4.

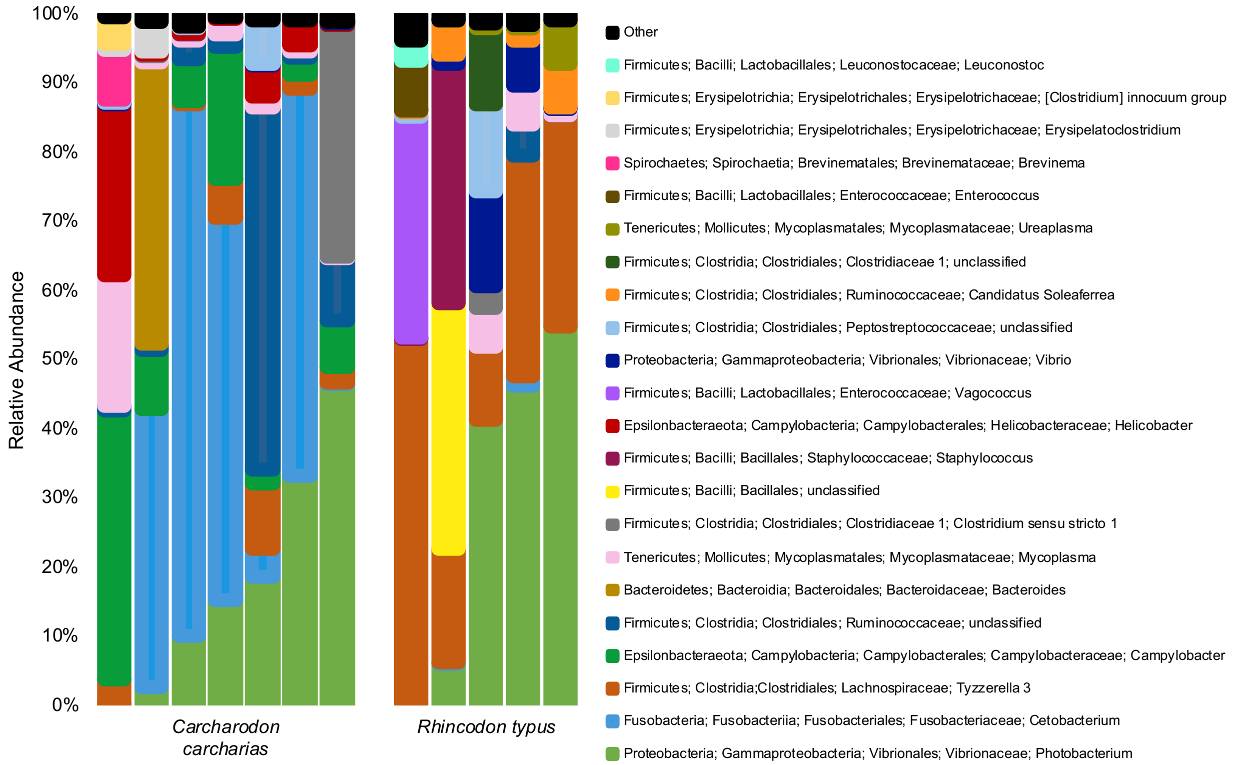

Supplemental Figure 2. The fecal microbial community composition of *Carcharodon carcharias* and *Rhincodon typus*, shown at the bacterial species level.

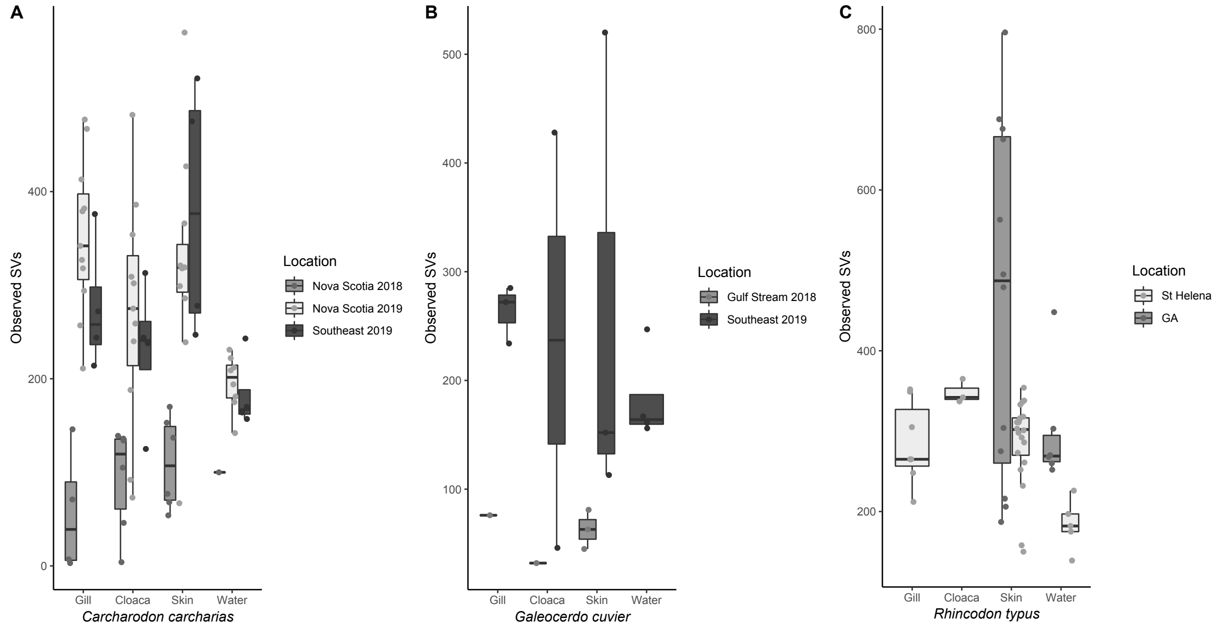

Supplemental Figure 3. Observed SVs for *Carcharodon carcharias* (panel A), *Galeocerdo cuvier* (panel B) and *Rhincodon typus* (panel C) for each body site and surrounding water at each location.

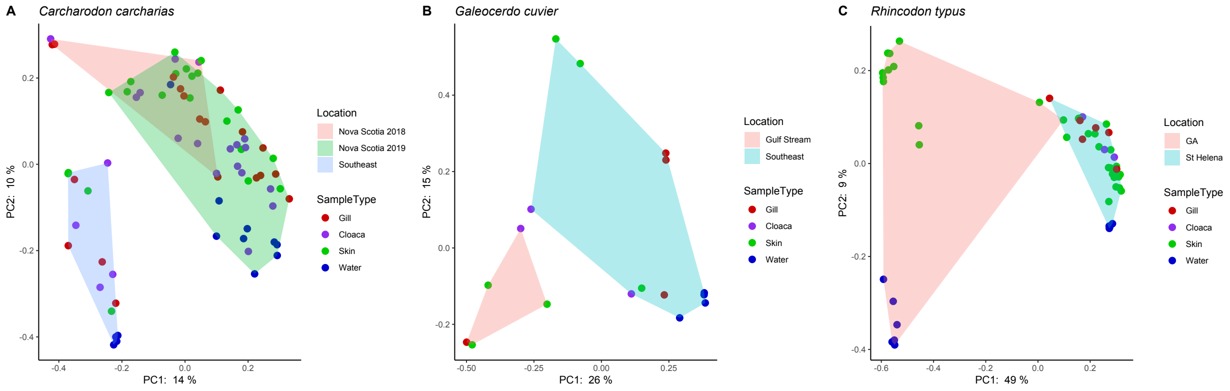

Supplemental Figure 4. Principal component analysis (PCoA) using Bray-Curtis dissimilarity and separated by shark species (*Carcharodon carcharias* (panel A), *Galeocerdo cuvier* (panel B), and *Rhincodon typus* (panel C)).

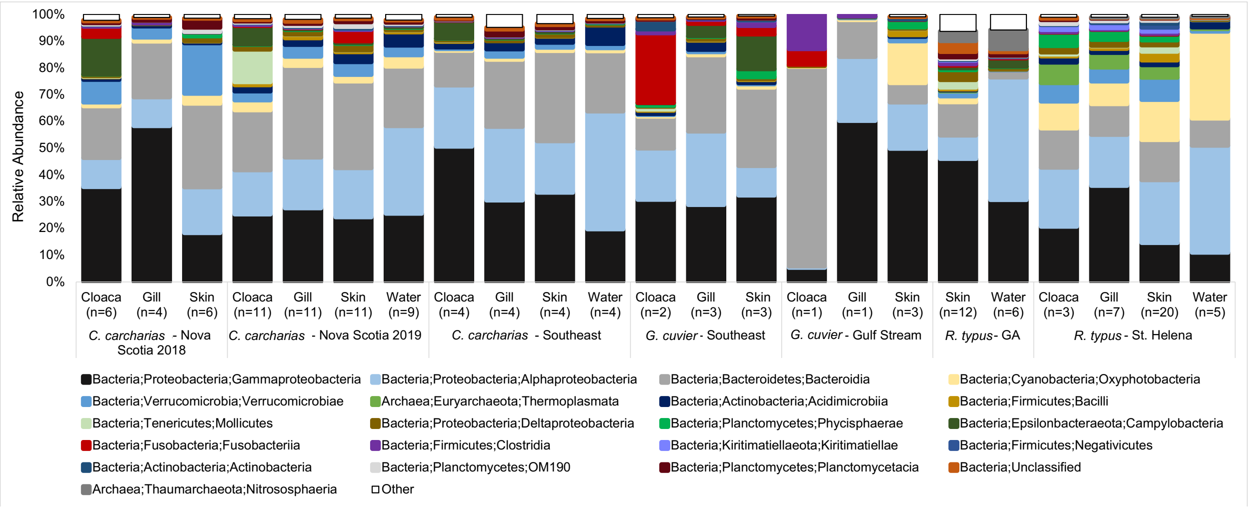

Supplemental Figure 5. Mean relative abundance of major microbial taxa among sample types. The number of each samples used for averaging is given in parentheses. Shark species include *Carcharodon carcharias*, *Galeocerdo cuvier,* and *Rhincodon typus*.

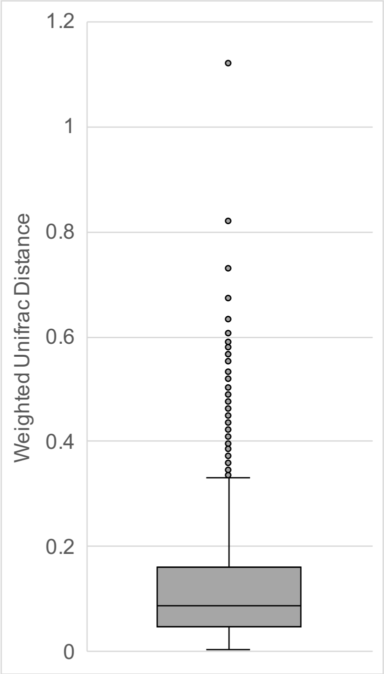

Supplemental Figure 6. Distribution of weighted UniFrac distances between PCR replicates. Box plot displays the median (center line line), 25th-75th percentiles, and whiskers showing a maximum 1.5 x IQR (interquartile range) All outlier replicates (in the fourth quartile, indicated by circles) were removed.

Supplemental Table 1. Number of samples collected from each species, location, and date. Further metadata for all OCEARH collected samples (*Galeocerdo cuvier* and *Carcharodon carcharias*) are given in Supplemental Table 2. n represents the total number of individual sharks represented in each category.

**Location**	**Species**	**n**	**Samples Collected**	**Dates**
Gulf Stream	*Galeocerdo cuvier* (tiger shark)	3	Dorsal skin (3), gill (3), and cloaca (1)	June, 2018
Nova Scotia	*Carcharodon carcharias* (white shark)	7	Dorsal skin (7), gill (4), cloaca (6), fecal (1), water (1)	September, 2018
U.S South East Coast	*Galeocerdo cuvier* (tiger shark)	4	Dorsal skin (3), gill (3), cloaca (2), water (4)	February, 2019
	Carcharodon carcharias (white shark)	4	Dorsal skin (4), gill (2), cloaca (4), fecal (1), water (4)	
Nova Scotia	*Carcharodon carcharias* (white shark)	11	Dorsal skin (11), gill (11), cloaca (11), fecal (5), water (8)	September, 2019
Georgia Aquarium	*Rhincodon typus* (whale shark)	4	Dorsal Pit (18), water (6)	6 time points between May 2019-Oct 2019
Maldives	*Rhincodon typus* (whale shark)	1	Fecal (1)	2015
Tanzania	*Rhincodon typus* (whale shark)	3	Fecal (3)	December 6, 2012; December 20, 2012; November 19, 2014
St. Helena	*Rhincodon typus* (whale shark)	29	Dorsal skin (29), gill (7), cloaca (3), fecal (1), water (5)	January-March, 2019

Supplemental Table 2. Metadata for all sharks and samples collected by OCEARCH.

Genus and Species	Expedition	Shark ID	Name	Sex	Maturity	Pre-caudal Length (cm)	Fork Length (cm)	Total Stretch Length (cm)	Estimated Weight (kg)	Girth (cm)
Galeocerdo cuvier	Gulf Stream 2018	GS2018-E4	Milt	F	Subadult	181	204	Not measured	85.7	80
Galeocerdo cuvier	Gulf Stream 2018	GS2018-E5	Conrad	M	Adult	243	265	Not measured	201	152
Galeocerdo cuvier	Gulf Stream 2018	GS2018-E6	Demott	M	Adult	266	292	Not measured	275.9	180
Carcharodon carcharias	Nova Scotia 2018	NS2018-01	Nova	M	Adult	285	319	Not measured	401	213
Carcharodon carcharias	Nova Scotia 2018	NS2018-02	Jefferson	M	Adult	323	365	Not measured	607.6	250
Carcharodon carcharias	Nova Scotia 2018	NS2018-03	Hal	M	Adult	315	352	400	543.3	230
Carcharodon carcharias	Nova Scotia 2018	NS2018-04	Shorty	M	Subadult	278	308	344	359.9	186
Carcharodon carcharias	Nova Scotia 2018	NS2018-05	Cabot	M	Subadult	231	260	296	213.4	164
Carcharodon carcharias	Nova Scotia 2018	NS2018-06	Jane	F	Subadult	239	269	305	237	155
Carcharodon carcharias	Nova Scotia 2018	NS2018-07	Luna	F	Adult	350	399	458	799.72	265
Galeocerdo cuvier	South East 2019	SE2019-01	Lando	M	Adult	246	269	306	211.1	128
Carcharodon carcharias	South East 2019	SE2019-02	Miss May	F	Subadult	276	309	311	363.5	189
Carcharodon carcharias	South East 2019	SE2019-03	Helena	F	Subadult	324	363	379	597.4	272
Carcharodon carcharias	South East 2019	SE2019-04	Brunswick	M	Subadult	223	253	266	196.16	148
Carcharodon carcharias	South East 2019	SE2019-05	Caroline	F	Subadult	221	366	388	612.8	233
Galeocerdo cuvier	South East 2019	SE2019-06	Not named	F	Adult	Not measured	Not measured	Not measured	Not measured	Not measured
Galeocerdo cuvier	South East 2019	SE2019-07	Not named	F	Adult	274.3	304.8	351.7	317.3	Not measured
Galeocerdo cuvier	South East 2019	SE2019-08	Not named	F	Subadult	Not measured	Not measured	Not measured	Not measured	Not measured
Carcharodon carcharias	Nova Scotia 2019	NS2019-01	Sydney	M	Adult	300	345	371	511	229
Carcharodon carcharias	Nova Scotia 2019	NS2019-02	Murdoch	M	Adult	322	364	393	602.5	211
Carcharodon carcharias	Nova Scotia 2019	NS2019-03	Unama'ki	F	Adult	370	421	470	943.7	273
Carcharodon carcharias	Nova Scotia 2019	NS2019-04	Caper	F	Subadult	204	236	265	158.3	140
Carcharodon carcharias	Nova Scotia 2019	NS2019-05	Bluenose	F	Subadult	273	301	353	335	183
Carcharodon carcharias	Nova Scotia 2019	NS2019-06	Ferg	M	Adult	281	318	356	397	266
Carcharodon carcharias	Nova Scotia 2019	NS2019-07	Shaw	M	Subadult	242	276	312	257	173
Carcharodon carcharias	Nova Scotia 2019	NS2019-08	Scotia	F	Subadult	265	303	337	342	183
Carcharodon carcharias	Nova Scotia 2019	NS2019-09	Ironbound	M	Adult	294	332	377	454	220
Carcharodon carcharias	Nova Scotia 2019	NS2019-10	Teazer	M	Subadult	258	289	327	296	185
Carcharodon carcharias	Nova Scotia 2019	NS2019-11	Vimy	M	Adult	309	349	384	529	255

Supplemental Table 3. Contig assembly statistics from one *Rhincodon typus* and seven *Carcharodon carcharias* fecal samples using SPAdes 3.13.0 and the metaspades.py script, and quality assessed by Quast 5.0.2.

Genus and Species	Metagenome	Raw reads after QC	Largest Contig	N50	# Contigs
Carcharodon carcharias	NS201805	15,968,373	504,235	1,424	37,563
Carcharodon carcharias	SE201903	9,276,515 paired-end 3,496,507 long-read	171,262	15,153	5,635
Carcharodon carcharias	NS201903	6,549,422	120,877	5,326	10,081
Carcharodon carcharias	NS201906	7,861,499	163,551	1,264	32,778
Carcharodon carcharias	NS201908	6,783,369	441,016	3,539	14,470
Carcharodon carcharias	NS201910	6,026,496	110,496	647	97,151
Carcharodon carcharias	NS201911	5,521,299	215,586	5,105	19,731
Rhincodon typus	StHelena34	7,881,512	225,615	842	45,674

Supplemental Table 4. Metagenome assembled genomes (MAGs) from one *Rhincodon typus* and seven *Carcharodon carcharias* fecal samples. Contigs (>5,000 bp) were binned using MaxBin2.0. Taxonomy and quality were estimated using MiGA. MAGs with quality less than 25.5 are grayed out. Short reads were mapped back to MAGs using Bowtie2 to determine percent alignment for each MAG.
**Photobacterium damselae* bins were merged for these metagenomes.

Species	MAG	Taxonomy	p-value	Complete-ness	Contamination	Quality	Percent mapped
R. typus	StHelena34_006	Clostridia	0.003	58.5	2.8	44.5	1.16%
R. typus	StHelena34_005	Mollicutes	0.134	92.5	28.3	-49.0	0.92%
R. typus	StHelena34_004	Clostridia	0.004	94.3	3.8	75.3	1.69%
R. typus	StHelena34_003	Bacteria	0.0	0.9	0	0.9	2.01%
R. typus	StHelena34_002	Mollicutes	0.051	44.3	4.7	20.8	3.47%
R. typus	StHelena34_001	*Photobacterium damselae*	0.052	99.1	23.6	-18.9	29.80%
C. carcharias	SE201903_007	Clostridiales	0.449	84.9	2.8	70.9	0.11%
C. carcharias	SE201903_006	Clostridium	0.248	80.2	7.5	42.7	0.74%
C. carcharias	SE201903_005	Campylobacteraceae	0.245	89.6	42.5	-122.9	1.07%
C. carcharias	SE201903_004	Fusobacterium	0.395	98.1	1.9	88.6	1.00%
C. carcharias	SE201903_003	*Photobacterium damselae*	0.007	13.2	0.9	8.7	37.02
C. carcharias	SE201903_001_002*	*Photobacterium damselae*	0.012	85.6	2.7	72.1	45.75%
C. carcharias	NS20198_005	*Photobacterium damselae*	0.002	68.9	23.6	-49.6	0.44%
C. carcharias	NS20198_004	Erysipelotrichaceae	0.006	84.0	16.0	4	1.18%
C. carcharias	NS20198_003	Campylobacteraceae	0.239	73.6	23.6	-44.4	1.92%
C. carcharias	NS20198_002	Fusobacterium	0.410	82.1	1.9	72.6	2.22%
C. carcharias	NS20198_001	*Bacteroides fragilis*	0.001	98.1	5.7	69.6	65.24%
C. carcharias	NS20196_007	Clostridia	0.003	80.2	58.5	-212.3	0.83%
C. carcharias	NS20196_006	Campylobacteraceae	0.27	81.1	16	1.1	1.68%
C. carcharias	NS20196_005	Fusobacteriaceae	0.256	72.6	5.7	44.1	6.29%
C. carcharias	NS20196_004	*Photobacterium damselae*	0.022	16.0	0	16.0	12.91%
C. carcharias	NS20196_003	Campylobacterales	0.380	93.4	0.9	88.9	9.59%
C. carcharias	NS20196_002	*Photobacterium damselae*	0.000	84.0	24.5	-38.5	11.3%
C. carcharias	NS20196_001	Bacteria	0.000	0.9	0	0.9	11.0%
C. carcharias	NS20193_005	Erysipelotrichaceae	0.049	88.7	40.6	-114.3	1.19%
C. carcharias	NS20193_004	Firmicutes	0.493	98.1	78.3	-293.4	3.55%
C. carcharias	NS20193_003	Campylobacteraceae	0.371	69.8	34.9	-104.7	6.21%
C. carcharias	NS20193_002	Campylobacterales	0.336	59.4	1.9	49.9	10.19%
C. carcharias	NS20193_001	Campylobacterales	0.391	97.2	2.8	83.2	36.19%
C. carcharias	NS201911_007	Campylobacterales	0.449	93.4	0.9	88.9	0.65%
C. carcharias	NS201911_006	Clostridia	0.035	84.9	34.9	-89.6	1.39%
C. carcharias	NS201911_005	Clostridiales	0.383	82.1	17.9	-7.4	3.49%

C. carcharias	NS201911_004	*Photobacterium damselae*	0.003	100	6.6	67.0	16.86%
C. carcharias	NS201911_003	Fusobacteriaceae	0.333	58.5	0.9	54.0	11.13%
C. carcharias	NS201911_002	Campylobacteraceae	0.324	100.0	9.4	53.0	27.83%
C. carcharias	NS201911_001	Fusobacteriia	0.235	25.5	0	25.5	6.76%
C. carcharias	NS201910_004	Campylobacterales	0.493	86.8	12.3	25.3	0.85%
C. carcharias	NS201910_003	*Photobacterium damselae*	0.002	100	24.5	-22.5	3.52%
C. carcharias	NS201910_002	Campylobacteraceae	0.266	75.5	0.9	71	1.08%
C. carcharias	NS201910_001	Fusobacterium	0.038	93.4	1.9	83.9	7.51%
C. carcharias	NS201805_007	Campylobacteraceae	0.463	77.5	38.7	-116	0.80%
C. carcharias	NS201805_006	Vibrionales	0.426	94.6	42.3	-116.9	2.84%
C. carcharias	NS201805_005	Clostrida	0.013	77.5	20.7	-26	0.86%
C. carcharias	NS201805_004	Campylobacteraceae	0.238	76.6	0.9	72.1	1.91%
C. carcharias	NS201805_003	Fusobacteriaceae	0.054	82.9	1.8	73.9	2.27%
C. carcharias	NS201805_001_002*	*Photobacterium damselae*	0.002	95.5	3.6	77.5	46.84%

Supplemental Table 5. Species used for fecal microbiome comparisons (Fig. 6). For sharks and marine mammals, *toothed or †filter-feeding strategies are noted accordingly.

Genus and Species	n	Common Name	Diet	Data Source
Balaenoptera borealis	1	Sei Whale	Carnivore (marine-mammal)†	Sanders et al., 2015
Carcharadon carcharias	7	White Shark	Carnivore (shark) †	This study
Centropyge flavissima	4	Lemon-peel Angelfish	Herbivore (teleost)	Pratte et al., 2018
Cephalopholis argus	4	Peacock Grouper	Carnivore (teleost)	Pratte et al., 2018
Delphinapterus leucas	2	Beluga Whale	Carnivore (marine-mammal)*	Sanders et al., 2015
Eubalaena glacialis	7	Right Whale	Carnivore (marine-mammal) †	Sanders et al., 2015
Hippopotamus amphibius	4	Hippo	Herbivore (terrestrial-mammal)	Sanders et al., 2015
Martes pennanti	1	Fisher-Cat	Carnivore (terrestrial-mammal)	Sanders et al., 2015
Megaptera novaeangliae	3	Humpback Whale	Carnivore (marine-mammal) †	Sanders et al., 2015
Odocoileus virginianus	1	White-tailed Deer	Herbivore (terrestrial-mammal)	Sanders et al., 2015
Oryctolagus cuniculus	1	Rabbit	Herbivore (terrestrial-mammal)	Sanders et al., 2015
Paracirrhites arcatus	11	Arc-eye Hawkfish	Carnivore (teleost)	Pratte et al., 2018
Pterois radiata	2	Lionfish	Carnivore (teleost)	Pratte et al., 2018
Rhincodon typus	5	Whale Shark	Carnivore (marine-mammal) †	This study
Scarus psittacus	3	Pale-nose Parrotfish	Herbivore (teleost)	Pratte et al., 2018
Stegastes nigricans	26	Farmer Fish	Herbivore (teleost)	Pratte et al., 2018
Tursiops truncatus	2	Bottlenose Dolphin	Carnivore (marine-mammal)*	Sanders et al., 2015

Supplemental Table 6. PERMANOVA calculated from weighted UniFrac distances from the OCEARCH Nova Scotia 2019 expedition only. Global parameters; n=41, groups=4, t=3.31, p-value=0.001, and permutations=999. Bold indicates significant adjust p-values (q) ≤ 0.01.

Group 1	*Group 2*	*Sample size*	*pseudo-F*	*p-value*	*q-value*
**Cloaca**	**Gill**	22	2.752	**0.005**	**0.0075**
**Cloaca**	**Skin**	22	2.123	0.024	0.0288
**Cloaca**	**Water**	19	3.049	**0.004**	**0.0075**
**Gill**	**Skin**	22	0.886	0.556	0.5560
**Gill**	**Water**	19	9.784	**0.001**	**0.0030**
**Skin**	**Water**	19	6.350	**0.001**	**0.0030**

Supplemental Table 7. The Southeast OCEARCH expedition was the only location from which multiple species (*Carcharodon carcharias,* and *Galeocerdo cuvier*) were sampled. Within the same body site (gill, cloaca, and skin), 139 sequence variants (SVs) were significantly different between shark species, 41 associated with the gills, 53 associated with the cloaca, and 45 associated with the skin. Significantly different SVs were determined using DESeq2.

log2 Fold Change	Adjusted p-value	Body site	Species with high abundance	Phylum	Class	Order	Family	Genus	Species
26.86	8.40E-06	Gill	G. cuvier	Actinobacteria	Actinobacteria	Corynebacteriales	Dietziaceae	Dietzia	
29.63	1.63E-07	Skin	G. cuvier	Actinobacteria	Actinobacteria	Corynebacteriales	Dietziaceae	Dietzia	
31.94	3.99E-08	Gill	G. cuvier	Actinobacteria	Actinobacteria	Micrococcales	Micrococcaceae	Micrococcus	
35.43	2.37E-10	Skin	G. cuvier	Actinobacteria	Actinobacteria	Micrococcales	Micrococcaceae	Micrococcus	
27.85	3.48E-06	Gill	C. carcharias	Bacteroidetes	Bacteroidia	Chitinophagales	Saprospiraceae		
29.18	1.04E-06	Gill	C. carcharias	Bacteroidetes	Bacteroidia	Chitinophagales	Saprospiraceae		
36.64	2.39E-10	Gill	C. carcharias	Bacteroidetes	Bacteroidia	Chitinophagales	Saprospiraceae	Aureispira	CNJ640
27.03	2.53E-06	Skin	C. carcharias	Bacteroidetes	Bacteroidia	Chitinophagales	Saprospiraceae		
27.78	1.40E-06	Skin	C. carcharias	Bacteroidetes	Bacteroidia	Chitinophagales	Saprospiraceae		
32.79	6.79E-09	Skin	C. carcharias	Bacteroidetes	Bacteroidia	Chitinophagales	Saprospiraceae		
22.45	0.0018	Cloaca	C. carcharias	Bacteroidetes	Bacteroidia	Cytophagales	Cyclobacteriaceae	Cyclobacterium	
24.12	0.00057	Cloaca	G. cuvier	Bacteroidetes	Bacteroidia	Cytophagales	Hymenobacteraceae	Hymenobacter	
22.60	0.0013	Cloaca	G. cuvier	Bacteroidetes	Bacteroidia	Cytophagales	Hymenobacteraceae	Hymenobacter	
25.91	6.64E-06	Skin	G. cuvier	Bacteroidetes	Bacteroidia	Cytophagales	Hymenobacteraceae	Hymenobacter	
28.62	1.87E-06	Gill	C. carcharias	Bacteroidetes	Bacteroidia	Flavobacteriales	Flavobacteriaceae	Tenacibaculum	
18.25	0.020	Cloaca	C. carcharias	Bacteroidetes	Bacteroidia	Flavobacteriales	Flavobacteriaceae	NS4 marine group	
18.40	0.018	Cloaca	C. carcharias	Bacteroidetes	Bacteroidia	Flavobacteriales	Cryomorphaceae		
19.76	0.0088	Cloaca	C. carcharias	Bacteroidetes	Bacteroidia	Flavobacteriales	Flavobacteriaceae		
19.82	0.0088	Cloaca	C. carcharias	Bacteroidetes	Bacteroidia	Flavobacteriales	NS9 marine group		
19.86	0.00039	Cloaca	C. carcharias	Bacteroidetes	Bacteroidia	Flavobacteriales	Flavobacteriaceae	NS4 marine group	
19.96	0.0082	Cloaca	C. carcharias	Bacteroidetes	Bacteroidia	Flavobacteriales	Flavobacteriaceae	NS5 marine group	
21.01	0.00094	Cloaca	C. carcharias	Bacteroidetes	Bacteroidia	Flavobacteriales	Flavobacteriaceae	Tenacibaculum	
21.73	0.0029	Cloaca	C. carcharias	Bacteroidetes	Bacteroidia	Flavobacteriales	Flavobacteriaceae	NS5 marine group	
22.38	4.78E-05	Cloaca	C. carcharias	Bacteroidetes	Bacteroidia	Flavobacteriales	Flavobacteriaceae	Mesonia	
24.34	1.04E-08	Cloaca	C. carcharias	Bacteroidetes	Bacteroidia	Flavobacteriales	Flavobacteriaceae	Muricauda	
19.64	0.0012	Skin	C. carcharias	Bacteroidetes	Bacteroidia	Flavobacteriales	Flavobacteriaceae	NS5 marine group	
20.58	0.00058	Skin	C. carcharias	Bacteroidetes	Bacteroidia	Flavobacteriales	Cryomorphaceae		
21.48	0.00028	Skin	C. carcharias	Bacteroidetes	Bacteroidia	Flavobacteriales	Flavobacteriaceae	NS5 marine group	
21.58	0.00027	Skin	C. carcharias	Bacteroidetes	Bacteroidia	Flavobacteriales	Flavobacteriaceae	NS4 marine group	
27.16	2.43E-06	Skin	C. carcharias	Bacteroidetes	Bacteroidia	Flavobacteriales	Flavobacteriaceae	Tenacibaculum	
35.41	2.79E-10	Skin	C. carcharias	Bacteroidetes	Bacteroidia	Flavobacteriales	Flavobacteriaceae	Tenacibaculum	
28.12	2.28E-06	Gill	G. cuvier	Bacteroidetes	Bacteroidia	Flavobacteriales	Flavobacteriaceae		
27.97	2.69E-06	Gill	G. cuvier	Bacteroidetes	Bacteroidia	Flavobacteriales	Flavobacteriaceae	NS4 marine group	
25.92	2.05E-05	Gill	G. cuvier	Bacteroidetes	Bacteroidia	Flavobacteriales	Flavobacteriaceae	NS5 marine group	
21.14	0.0013	Gill	G. cuvier	Bacteroidetes	Bacteroidia	Flavobacteriales			
30.61	2.29E-07	Gill	C. carcharias	Bacteroidetes	Bacteroidia	Sphingobacteriales			
29.91	1.62E-07	Skin	C. carcharias	Bacteroidetes	Bacteroidia	Sphingobacteriales			
20.49	0.00094	Cloaca	C. carcharias	Bacteroidetes	Rhodothermia	Balneolales	Balneolaceae	Balneola	
25.02	5.37E-05	Gill	C. carcharias	Chloroflexi	Anaerolineae	SBR1031	A4b		
28.32	2.28E-06	Gill	C. carcharias	Chloroflexi	Anaerolineae	SBR1031	A4b		
28.52	1.96E-06	Gill	C. carcharias	Chloroflexi	Anaerolineae	SBR1031	A4b		
33.65	7.73E-09	Gill	C. carcharias	Chloroflexi	Anaerolineae	SBR1031	A4b		
20.26	4.78E-05	Cloaca	C. carcharias	Chloroflexi	Anaerolineae	SBR1031	A4b		
25.21	1.12E-05	Skin	C. carcharias	Chloroflexi	Anaerolineae	SBR1031	A4b		
26.39	4.15E-06	Skin	C. carcharias	Chloroflexi	Anaerolineae	SBR1031	A4b		
27.26	2.26E-06	Skin	C. carcharias	Chloroflexi	Anaerolineae	SBR1031	A4b		
25.66	0.00028	Cloaca	C. carcharias	Epsilonbacteraeota	Campylobacteria	Campylobacterales	Campylobacteraceae	Campylobacter	
39.49	2.76E-12	Gill	G. cuvier	Epsilonbacteraeota	Campylobacteria	Campylobacterales	Arcobacteraceae	Arcobacter	
39.96	1.00E-12	Skin	G. cuvier	Epsilonbacteraeota	Campylobacteria	Campylobacterales	Arcobacteraceae	Arcobacter	
34.62	2.35E-09	Gill	G. cuvier	Firmicutes	Bacilli	Lactobacillales	Enterococcaceae	Vagococcus	
17.28	0.029	Cloaca	G. cuvier	Firmicutes	Bacilli	Lactobacillales	Enterococcaceae	Vagococcus	
35.44	2.37E-10	Skin	G. cuvier	Firmicutes	Bacilli	Lactobacillales	Enterococcaceae	Vagococcus	
27.39	8.69E-05	Cloaca	G. cuvier	Firmicutes	Clostridia	Clostridiales	Lachnospiraceae		
32.62	1.77E-08	Gill	G. cuvier	Fusobacteria	Fusobacteriia	Fusobacteriales	Fusobacteriaceae	Fusobacterium	
22.18	1.78E-05	Gill	G. cuvier	Fusobacteria	Fusobacteriia	Fusobacteriales	Fusobacteriaceae	Cetobacterium	

14.31	0.030	Cloaca	G. cuvier	Fusobacteria	Fusobacteriia	Fusobacteriales	Fusobacteriaceae	Cetobacterium	
34.35	7.27E-10	Skin	G. cuvier	Fusobacteria	Fusobacteriia	Fusobacteriales	Fusobacteriaceae	Fusobacterium	
41.35	2.15E-13	Gill	G. cuvier	Planctomycetes	Phycisphaerae	Phycisphaerales	Phycisphaeraceae	CL500-3	
17.81	0.021	Cloaca	G. cuvier	Planctomycetes	Phycisphaerae	Phycisphaerales	Phycisphaeraceae	CL500-3	
22.56	0.00046	Gill	C. carcharias	Planctomycetes	Planctomycetacia	Planctomycetales	Gimesiaceae	Gimesia	
21.02	0.0043	Cloaca	C. carcharias	Planctomycetes	Planctomycetacia	Planctomycetales	Gimesiaceae	Gimesia	
36.49	1.18E-10	Skin	G. cuvier	Planctomycetes	Planctomycetacia	Planctomycetales	Rubinisphaeraceae		
31.11	1.35E-07	Gill	C. carcharias	Proteobacteria	Alphaproteobacteria	Caulobacterales	Hyphomonadaceae	JNU-L072	
28.38	7.69E-07	Skin	C. carcharias	Proteobacteria	Alphaproteobacteria	Caulobacterales	Hyphomonadaceae		
8.63	0.0063	Cloaca	C. carcharias	Proteobacteria	Alphaproteobacteria	Parvibaculales	Parvibaculaceae		
9.59	0.021	Cloaca	C. carcharias	Proteobacteria	Alphaproteobacteria	Puniceispirillales	SAR116 clade		
21.08	0.0014	Gill	G. cuvier	Proteobacteria	Alphaproteobacteria	Rhizobiales	Beijerinckiaceae	Methylobacterium	
20.60	0.00053	Skin	G. cuvier	Proteobacteria	Alphaproteobacteria	Rhizobiales	Beijerinckiaceae	Methylobacterium	
29.28	9.79E-07	Gill	C. carcharias	Proteobacteria	Alphaproteobacteria	Rhodobacterales	Rhodobacteraceae		
20.98	0.0011	Cloaca	C. carcharias	Proteobacteria	Alphaproteobacteria	Rhodobacterales	Rhodobacteraceae		
29.64	1.89E-07	Skin	C. carcharias	Proteobacteria	Alphaproteobacteria	Rhodobacterales	Rhodobacteraceae		
19.51	0.0099	Cloaca	C. carcharias	Proteobacteria	Alphaproteobacteria	Rickettsiales	S25-593		
25.67	2.51E-05	Gill	G. cuvier	Proteobacteria	Alphaproteobacteria	Rickettsiales	S25-593		
25.87	6.72E-06	Skin	C. carcharias	Proteobacteria	Alphaproteobacteria	Sphingomonadales	Sphingomonadaceae	Sphingomonas	
30.19	3.01E-07	Gill	G. cuvier	Proteobacteria	Alphaproteobacteria	Sphingomonadales	Sphingomonadaceae	Sphingomonas	
21.69	0.00087	Gill	G. cuvier	Proteobacteria	Alphaproteobacteria	Sphingomonadales	Sphingomonadaceae		
23.80	0.00063	Cloaca	G. cuvier	Proteobacteria	Alphaproteobacteria	Sphingomonadales	Sphingomonadaceae		
22.55	0.0013	Cloaca	G. cuvier	Proteobacteria	Alphaproteobacteria	Sphingomonadales	Sphingomonadaceae	Sphingomonas	
27.07	2.26E-06	Skin	G. cuvier	Proteobacteria	Alphaproteobacteria	Sphingomonadales	Sphingomonadaceae		
23.11	6.44E-05	Skin	G. cuvier	Proteobacteria	Alphaproteobacteria	Sphingomonadales	Sphingomonadaceae		
21.64	0.0030	Cloaca	C. carcharias	Proteobacteria	Alphaproteobacteria	uncultured			
22.91	0.00036	Gill	C. carcharias	Proteobacteria	Alphaproteobacteria				
23.33	0.00025	Gill	C. carcharias	Proteobacteria	Alphaproteobacteria				
18.92	0.014	Cloaca	C. carcharias	Proteobacteria	Alphaproteobacteria				
21.91	0.00020	Skin	C. carcharias	Proteobacteria	Alphaproteobacteria				
30.57	8.08E-08	Skin	C. carcharias	Proteobacteria	Deltaproteobacteria	NB1-j			
10.24	0.00027	Cloaca	C. carcharias	Proteobacteria	Deltaproteobacteria	SAR324 clade (Marine group B)			
19.14	0.0090	Cloaca	C. carcharias	Proteobacteria	Gammaproteobacteria	Acidithiobacillales	Acidithiobacillaceae	KCM-B-112	
23.32	2.85E-05	Cloaca	C. carcharias	Proteobacteria	Gammaproteobacteria	Acidithiobacillales	Acidithiobacillaceae	KCM-B-112	
22.82	0.00039	Gill	C. carcharias	Proteobacteria	Gammaproteobacteria	Alteromonadales	Idiomarinaceae	Idiomarina	
23.49	0.00022	Gill	C. carcharias	Proteobacteria	Gammaproteobacteria	Alteromonadales	Marinobacteraceae	Marinobacter	
17.36	0.031	Cloaca	C. carcharias	Proteobacteria	Gammaproteobacteria	Alteromonadales	Shewanellaceae	Shewanella	
21.39	0.0035	Cloaca	C. carcharias	Proteobacteria	Gammaproteobacteria	Alteromonadales	Idiomarinaceae	Idiomarina	
23.15	0.0012	Cloaca	C. carcharias	Proteobacteria	Gammaproteobacteria	Alteromonadales	Shewanellaceae	Shewanella	
21.91	0.00020	Skin	C. carcharias	Proteobacteria	Gammaproteobacteria	Alteromonadales	Shewanellaceae	Shewanella	
23.49	5.49E-05	Skin	C. carcharias	Proteobacteria	Gammaproteobacteria	Alteromonadales	Idiomarinaceae	Idiomarina	
23.54	5.41E-05	Skin	C. carcharias	Proteobacteria	Gammaproteobacteria	Alteromonadales	Marinobacteraceae	Marinobacter	
40.42	1.98E-17	Gill	G. cuvier	Proteobacteria	Gammaproteobacteria	Alteromonadales	Shewanellaceae	Shewanella	
26.61	1.01E-05	Gill	G. cuvier	Proteobacteria	Gammaproteobacteria	Alteromonadales	Shewanellaceae	Shewanella	
25.55	0.00027	Cloaca	G. cuvier	Proteobacteria	Gammaproteobacteria	Alteromonadales	Shewanellaceae	Shewanella	
24.13	6.61E-05	Cloaca	G. cuvier	Proteobacteria	Gammaproteobacteria	Alteromonadales	Shewanellaceae	Shewanella	
17.27	0.00027	Cloaca	G. cuvier	Proteobacteria	Gammaproteobacteria	Alteromonadales	Psychromonadaceae	Psychromonas	
25.86	6.12E-06	Skin	G. cuvier	Proteobacteria	Gammaproteobacteria	Alteromonadales	Shewanellaceae	Shewanella	
33.65	7.73E-09	Gill	C. carcharias	Proteobacteria	Gammaproteobacteria	BD7-8			
21.28	0.0013	Gill	C. carcharias	Proteobacteria	Gammaproteobacteria	Cellvibrionales	Cellvibrionaceae	Marinagarivorans	
21.59	0.0010	Gill	C. carcharias	Proteobacteria	Gammaproteobacteria	Cellvibrionales	Cellvibrionaceae	Gilvimarinus	
24.25	0.00062	Cloaca	C. carcharias	Proteobacteria	Gammaproteobacteria	Cellvibrionales	Cellvibrionaceae	Gilvimarinus	
22.82	9.45E-05	Skin	C. carcharias	Proteobacteria	Gammaproteobacteria	Cellvibrionales	Cellvibrionaceae	Marinagarivorans	
23.46	5.49E-05	Skin	C. carcharias	Proteobacteria	Gammaproteobacteria	Cellvibrionales	Cellvibrionaceae	Gilvimarinus	
10.01	0.017	Skin	C. carcharias	Proteobacteria	Gammaproteobacteria	KI89A clade			
23.85	0.00016	Gill	C. carcharias	Proteobacteria	Gammaproteobacteria	Oceanospirillales	Alcanivoracaceae	Alcanivorax	
19.95	0.0082	Cloaca	C. carcharias	Proteobacteria	Gammaproteobacteria	Oceanospirillales	SS1-B-06-26		
27.03	0.00014	Cloaca	C. carcharias	Proteobacteria	Gammaproteobacteria	Oceanospirillales	Alcanivoracaceae	Alcanivorax	
11.02	0.042	Skin	C. carcharias	Proteobacteria	Gammaproteobacteria	Oceanospirillales	Halomonadaceae	Halomonas	
18.98	0.0021	Skin	C. carcharias	Proteobacteria	Gammaproteobacteria	Oceanospirillales	SS1-B-06-26		
23.88	3.19E-06	Skin	C. carcharias	Proteobacteria	Gammaproteobacteria	Oceanospirillales	Saccharospirillaceae	Oleispira	Oleispira
25.28	1.07E-05	Skin	C. carcharias	Proteobacteria	Gammaproteobacteria	Oceanospirillales	Alcanivoracaceae	Alcanivorax	
32.73	1.72E-08	Gill	G. cuvier	Proteobacteria	Gammaproteobacteria	Oceanospirillales	Halomonadaceae	Oceanospirillum	
25.16	4.12E-05	Gill	G. cuvier	Proteobacteria	Gammaproteobacteria	Oceanospirillales	SS1-B-06-26		
20.57	4.78E-05	Cloaca	C. carcharias	Proteobacteria	Gammaproteobacteria	OM182 clade			
22.62	0.00045	Gill	C. carcharias	Proteobacteria	Gammaproteobacteria	Pseudomonadales	Moraxellaceae	Psychrobacter	
19.41	0.010	Cloaca	C. carcharias	Proteobacteria	Gammaproteobacteria	Pseudomonadales	Moraxellaceae	Psychrobacter	
22.64	0.00011	Skin	C. carcharias	Proteobacteria	Gammaproteobacteria	Pseudomonadales	Moraxellaceae	Psychrobacter	
17.02	0.00033	Cloaca	G. cuvier	Proteobacteria	Gammaproteobacteria	Pseudomonadales	Moraxellaceae	Psychrobacter	

20.94	3.19E-06	Skin	C. carcharias	Proteobacteria	Gammaproteobacteria	SAR86 clade	bacterium WHC7-12		
22.76	1.23E-09	Cloaca	C. carcharias	Proteobacteria	Gammaproteobacteria	Vibrionales	Vibrionaceae	Vibrio	
27.81	7.48E-05	Cloaca	G. cuvier	Proteobacteria	Gammaproteobacteria	Vibrionales	Vibrionaceae	Photobacterium	
27.43	8.69E-05	Cloaca	G. cuvier	Proteobacteria	Gammaproteobacteria	Vibrionales	Vibrionaceae	Vibrio	
26.28	0.00018	Cloaca	G. cuvier	Proteobacteria	Gammaproteobacteria	Vibrionales	Vibrionaceae	Vibrio	
25.49	0.00027	Cloaca	G. cuvier	Proteobacteria	Gammaproteobacteria	Vibrionales	Vibrionaceae	Photobacterium	
22.28	0.00061	Cloaca	C. carcharias	Proteobacteria	Gammaproteobacteria				
23.49	7.39E-06	Skin	C. carcharias	Proteobacteria	Gammaproteobacteria				
32.71	6.79E-09	Skin	C. carcharias	Proteobacteria	Gammaproteobacteria				
25.41	0.00027	Cloaca	G. cuvier	Tenericutes	Mollicutes	Mycoplasmatales	Mycoplasmataceae	Ureaplasma	
24.19	0.00057	Cloaca	G. cuvier	Tenericutes	Mollicutes	Mycoplasmatales	Mycoplasmataceae	Mycoplasma	Mycoplasma
33.65	7.73E-09	Gill	C. carcharias	Verrucomicrobia	Verrucomicrobiae	Opitutales	Puniceicoccaceae	Coraliomargarita	
20.50	0.00057	Cloaca	C. carcharias	Verrucomicrobia	Verrucomicrobiae	Opitutales	Puniceicoccaceae	Lentimonas	
20.64	1.86E-05	Skin	C. carcharias	Verrucomicrobia	Verrucomicrobiae	Opitutales	Puniceicoccaceae	Lentimonas	
32.06	1.38E-08	Skin	C. carcharias	Verrucomicrobia	Verrucomicrobiae	Opitutales	Puniceicoccaceae	Coraliomargarita	
